# Detection of unknown strawberry diseases based on OpenMatch and two-head network for continual learning

**DOI:** 10.3389/fpls.2022.989086

**Published:** 2022-09-15

**Authors:** Kan Jiang, Jie You, Ulzii-Orshikh Dorj, Hyongsuk Kim, Joonwhoan Lee

**Affiliations:** ^1^Department of Computer Science and Engineering, Artificial Intelligence Lab, Jeonbuk National University, Jeonju, South Korea; ^2^Division of Electronics and Information Engineering, Jeonbuk National University, Jeonju, South Korea; ^3^Core Research Institute of Intelligent Robots, Jeonbuk National University, Jeonju, South Korea

**Keywords:** continual learning, plant diseases, Open Set Recognition, Out-of-Distribution detection, two-head network, OpenMatch, strawberry disease classification

## Abstract

For continual learning in the process of plant disease recognition it is necessary to first distinguish between unknown diseases from those of known diseases. This paper deals with two different but related deep learning techniques for the detection of unknown plant diseases; Open Set Recognition (OSR) and Out-of-Distribution (OoD) detection. Despite the significant progress in OSR, it is still premature to apply it to fine-grained recognition tasks without outlier exposure that a certain part of OoD data (also called known unknowns) are prepared for training. On the other hand, OoD detection requires intentionally prepared outlier data during training. This paper analyzes two-head network included in OoD detection models, and semi-supervised OpenMatch associated with OSR technology, which explicitly and implicitly assume outlier exposure, respectively. For the experiment, we built an image dataset of eight strawberry diseases. In general, a two-head network and OpenMatch cannot be compared due to different training settings. In our experiment, we changed their training procedures to make them similar for comparison and show that modified training procedures resulted in reasonable performance, including more than 90% accuracy for strawberry disease classification as well as detection of unknown diseases. Accurate detection of unknown diseases is an important prerequisite for continued learning.

## Introduction

Plant disease monitoring is a critical means of improving productivity and enhancing crop quality. The traditional methods for diagnosis of plant diseases–visual analysis by a professional farmer or inspection of a sample in a laboratory–generally requires extensive professional knowledge and high costs. For this reason, an automated disease monitoring process will prove to be a valuable supplement to the labor and skill of farmers (Kim et al., 2021).

A number of research studies have applied deep learning techniques to automatic plant disease monitoring (Liu and Wang, 2021). However, most of the studies have been based on closed set recognition (CSR), which is prone to erroneous decisions when an unknown disease sample is detected because it must be classified into one of known classes. Moreover, discriminating images of plant diseases (or disorders) is a difficult task for computer vision, categorized into a fine-grained task involving both easy and hard problems.

In contrast, a human expert can naturally accumulate knowledge to improve their ability to accurately recognize plant diseases or disorders in an increasing number of categories. In order to program a machine to be similar to a human expert, it is necessary to continuously increase the amount of data and the number of categories it has access to. Open Set Recognition (OSR) and Out-of-Distribution (OoD) detection technology are used for continual machine learning and can be applied to plant disease recognition in order to differentiate unknown diseases and disorders from known diseases.

Generally speaking, for continual learning of open world tasks, both detection of unknown diseases and incremental active learning with unknowns should be addressed. However, unknowns need to be correctly identified before commencing active and incremental learning, so that their detection is essential to lifelong or continual learning for the performance of open world tasks.

**FIGURE 1:**
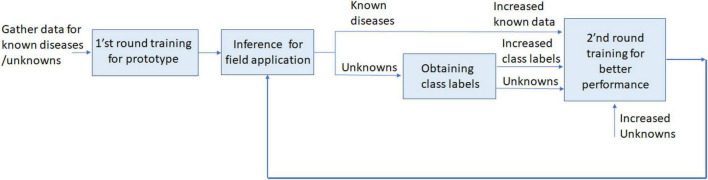
Unknown detection and continual learning for plant disease monitoring.

Figure 1 shows the continual learning process for plant disease monitoring. The unknowns should be identified in the inference stage, and then a second round of training is performed with additional known and unknown disease data, with (or without) increased number of categories.

Automatic detection of unknowns has been a traditional field of research (Scheirer et al., 2012) in computer vision and has recently received attention due to deep learning technology’s increasing popularity (Cardoso et al., 2015). In general, however, unknowns are not available in the learning process. In conventional CSR, the unknowns must be classified into a known class during the inference process, which degrades performance. To avoid such degradation, OSR should have a proper structure and be carefully trained.

There has been a large volume of research on OSR since it was formalized by Scheirer et al. (2012). Unfortunately, OSR technology in its current state is unable to be practically applied to fine-gained plant disease monitoring due to poor performance without assuming outlier (sometimes called known unknowns) exposure. OoD detection technology is closely related to OSR, but outliers can be partly assumed and prepared for training differently from OSR. In general, OoD detection encompasses all forms of distributional shift, while OSR specifically refers to semantic novelty (Vaze et al., 2021). However, in plant disease recognition based on image analysis, OSR is similar to the OoD detection when there is no severe distribution shift in captured image data, and a set of outlier data is assumed in the training (outlier exposure). Figure 1 assumes outlier exposure from the first round of training of the prototype model, because unknowns are incorporated with.

The goal of this paper is finding the practical solutions to detect unknowns for continual learning as shown in Figure 1, where a part of outliers is assumed to be prepared for training. For this purpose, the paper evaluates (Yu and Aizawa, 2019) a two-head network that uses OoD detection, and Saito et al. (2021) semi-supervised OpenMatch that uses OSR technology, both of which show reasonable performance for known plant disease recognition as well as unknown disease detection.

It is generally it is not appropriate to compare OoD and OSR because they require different settings for training. In order to change the semi-supervised OpenMatch into OoD detection similar to the two-head network, OpenMatch can be disassembled into two stages; one training stage to learn the One versus All (OVA) and softmax classifiers with labeled and OoD data, and another stage to learn the semi-supervised setting of OpenMatch with unlabeled samples including both inliers and outliers. Also, the two-head network can be retrained with FixMatch and fine-tuned after finding high confidence pseudo inliers and outliers from the inference process. After these modifications, the two different models can be comparable in terms of the performance for detecting and classifying both unknown and known diseases.

As shown in Figure 1, these two different modified models of OpenMatch and the two-head network are related with continual learning, because inliers and outliers of unknowns can be effectively recognized, and the results can be used for second round training to continuously improve the models’ performance.

The contributions of this paper can be summarized as follows:

1. The difficulty recognizing unknown plant diseases is related to continual learning and the progressive evolution of machine performance. We chose two different types of techniques, OpenMatch using OSR, and a two-head network using OoD detection, which are closely related technologies. To the best of our knowledge, this might be the first article to examine OSR and OoD detection for plant disease monitoring. In addition, this paper shows that OSR outlier exposure is a necessary assumption to adequately detect unknowns.
2. Open Set Recognition and Out-of-Distribution detection are difficult to compare. After the proper modifications, we compared OpenMatch with the two-head network to classify unknown strawberry diseases and related both classification models with continual learning. In addition, our results show that the contrastive regularization in FixMatch developed for semi-supervised OpenMatch was successfully applied to the two-head network to improve its performance.
3. We constructed an image dataset of strawberry diseases to validate OSR with assumed outlier exposure. The result of our experiment shows that both the two-head network and OpenMatch can provide reasonable performance for classifying the aforementioned eight strawberry diseases as well as detecting unknowns.

## Related works

In this section we summarize the use of OSR and OoD detection for continual learning and DNN-based plant disease monitoring.

### Open Set Recognition and Out-of-Distribution detection for continual learning

Recently, open world vision has received considerable attention in the field of computer vision, because it has the potential to resolve many realistic problems such as open set recognition, long-tailed distribution, and limited ontology of labels for life-long or continual learning (Open World Vision, 2021). Open world vision is also related to active or incremental learning because unknowns can be grouped to obtain labels, or should be learned with increased number of categories without catastrophic forgetting (Parisi et al., 2019).

An important task in open world vision is properly differentiating unknowns from known classes. In the inference phase of CSR, a sample should be classified into known classes included in the training phase. When using OSR, however, a classification model must be able to distinguish between the training classes, and indicate if an image comes from a class it has not yet encountered (Scheirer et al., 2012). This implies that unknowns are not exposed to the model during OSR training.

There are several types of deep learning-based OSR models. OpenMax (Bendale and Boult, 2016) is an extension of SoftMax that uses probability adapting Meta-Recognition concepts to activate patterns in the penultimate layer to recognize unknown. There are many generative models of OSR based on auto-encoders or GANs (Generalized Adversarial Networks). G (Generative)-OpenMax is an extension of OpenMax, in which unknown unknown class samples are artificially generated with GANs and are used for fine-tuning OpenMax (Ge et al., 2017). A class-conditioned Auto-Encoder for OSR is another kind of generative model in which an encoder/decoder model is used to classify known classes and unknowns (Oza and Patel, 2019). Outlier exposure is a necessary assumption to improve OCR performance, but there is a risk of overfitting, because only a limited amount of the voluminous outlier data is available for training. OpenGAN is the most recent generative model in which outlier exposure is assumed, but additional GANs are applied to supplement outlier data to prevent overfitting (Kong and Ramanan, 2021). OpenHybrid framework consists of an encoder to encode the input data into a joint embedding space, a classifier to classify samples to inlier classes, and a flow-based density estimator to detect whether a sample belongs to the unknown category (Zhang et al., 2020). There are many recent papers continuously being published with tutorials in OpenSetRecognition_list (2022).

While OSR is closely related to OoD detection (Hendrycks and Gimpel, 2016), OoD settings permit the use of additional data as examples of “OoD” data during training (Chen et al., 2021). Many deep leaning-based OoD detection methods have been developed. The maximum softmax probability is the simplest one to decide if something is an inlier or outlier. Generalized ODIN (Hsu et al., 2020), an extended version of ODIN, uses the decomposed confidence model, temperature scaling, and modified input preprocessing strategies (Liang et al., 2017). Also, many OoD detection methods were introduced by Salehi et al. (2021) including the two-head network that we consider in this paper.

The two-head network in the paper was published by Yu and Aizawa (2019) to find OoD samples. In plant disease monitoring, the set of OoD samples can included unknown diseases or disorders, as well as other images irrelevant to the task. When unknowns are included in OoD detection training data, they are called known unknowns. The set of OoD data prepared for training is a type of bias (Hsu et al., 2020), and reasonable OoD data should be chosen in two-head network training.

The OSR algorithm OpenMatch in the paper was released in 2021 (Saito et al., 2021), and an advanced modified version was published which added contrastive loss (Lee et al., 2022). The networks in OpenMatch are trained in a semi-supervised setting, which is different from OoD-based detection of the two-head network. However, semi-supervised learning can be treated as a method to expose outliers for training, because unlabeled data can include OoD samples as well as unlabeled inliers.

Due to (*a priori*) known unknowns in the training phase, it is hard to directly compare OoD detection with OSR. However, in practice, the distinction between OSR and OoD detection is not important if the outlier images are well prepared.

### Related works of deep learning-based plant disease monitoring

There are two types of deep learning models for plant disease monitoring: classification and deep object detection. The classification model can be used to find the name of a disease after an image is manually taken by a camera (Mohanty et al., 2016). In contrast, the deep object detection model can place the diseased area in a bounding box, so that it can be applied to automatic disease monitoring if the imaging apparatus is equipped with a mobile robot. There are excellent studies reported by Kim et al. (2021) and Liu and Wang (2021).

The following discussion focuses on the classification model, as we tried to apply said model to recognize the diseases with unknowns. In general, unknown object detection is a much more complicated task than object identification (Joseph et al., 2021).

There have been a number of deep neural network (DNN)-based classification approaches used to identify plant diseases and disorders. The DNN usually consists of a multilayer convolutional neural network (CNN)-based feature representation block (backbone), and a softmax classification block (head). Table 1 displays several selected applications of plant disease classification. The backbone network can be used depending on requirements of the applications. If fast recognition speed is required to scarify the accuracy, then a light DNN model like MobileNet may be a prudent choice (You and Lee, 2020). If the accuracy is more important than the speed, then a complex DNN backbone like ResNet might be optimal (He et al., 2016). There are numerous CNN-based off-the-shelf DNN backbones one can choose according to specific requirements (Tan and Le, 2019). A transformer-based backbone is another option to select as a DNN backbone (Dosovitskiy et al., 2020). Note that the backbone can be constructed to obtain better performance by including multiscale methods (Lin et al., 2017).

**TABLE 1:** Deep neural network (DNN)-based classification approaches for identification of plant diseases.

References	Network models	Dataset for pre-training	Plants	Dataset for fine-tuning	Disease classes
Barbedo, 2018	GoogleNet	ImageNet	12 spices		12
Ferentinos, 2018	AlexNet, GoogleNet, Overfeat, VGG16, AlexNetOWTBn		25 species	PlantVillage	58
Liu et al., 2017	AlexNet	ImageNet	apple	Collected from fields	4
Mukti and Biswas, 2019	AlexNet, VGG16,19, ResNet50	ImageNet	38 species	PlantVillage	38
Saleem et al., 2019	AlexNet, LeNet, VGG, GooLeNet, ResNet, DenseNet	ImageNet	38 species	PlantVillage	38
Kumar et al., 2020	ResNet34	ImageNet	14 species	New Plant Diseases Dataset	38
Rangarajan et al., 2018	AlexNet, VGG 16	ImageNet	7 species	Tomato crop	6
Aquil and Ishak, 2021	Vgg16,19, ResNet18,34,50,101, DenseNet120, SqueezeNet	PlantVillage	44 species	tomato leaves	9
Rao et al., 2022	VGG, ResNet based on Bi-CNN		38 species	PlantVillage	38
Rehman et al., 2022	MobileNetv2, DenseNet201	ImageNet	citrus	citrus diseases	6

The head structure of softmax classifiers is similar to each other, where the conditional probability distribution of class labels for given input image. Note that there might be multi-label classifiers which have more than one head. In this case, each separate head can be constructed using separate softmax classifiers to share the DNN backbone during multitasking and by sigmoid classifiers. In this paper, the *K*-OVA block in the OpenMatch structure has *K* separate softmax classifiers that share the backbone.

Transfer learning is widely used due to the lack of training data in many application areas, including plant disease monitoring, where a pre-trained backbone with a huge amount of data in the general domain is initialized to be fine-tuned in a specific application domain. For this purpose, a set of pre-trained parameters for the specific backbone model with an ImageNet dataset is available for constructing the classifier. However, the ImageNet dataset is so general that the domain-specific dataset such as LifeClEF 2017 might be the better choice for a backbone to be used for a specific application (Joly et al., 2017).

Many initial DNN-based plant disease monitoring systems were developed using the PlantVillage dataset which included a diverse group of crops. However, the success of DNN-based monitoring has resulted in diverse datasets built for various crops.

However, it is difficult to find previous research concerning the detection of unknown diseases, except cassava disease classification using CropNet (CropNet, 2020), where the network tried to classify four major cassava diseases on diseased leaves, normal leaves, and unknown. The detection technology of CropNet cannot be identified in detail, but presumably it is not a very complex algorithm.

**FIGURE 2:**
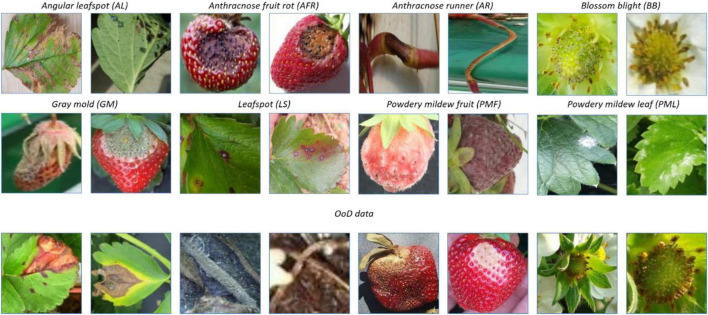
Prototypical images of known diseases and unknown diseases.

Meanwhile, there are more than 70 diseases and disorders introduced in Strawberry Diseases (2022), and it is difficult to paper sufficient data for all of them at once. Therefore, the probability of continual learning for detecting diseases and disorders increases with the increased number of classes and corresponding data. Figure 2 shows images of the 8 classes of known diseases and several unknown disorders. Note that the plant parts including fruit, leaves, runners, and flowers are easy to differentiate, while diseases of the same plant part are difficult to discern. As a result, the disease recognition task is fine-grained, having both easy and hard problems.

## Materials and methods

In this section, we introduce the two-head network (Yu and Aizawa, 2019) and OpenMatch (Saito et al., 2021) which were used in the experiments. We discuss how the two-head network can be implemented to recognize unknowns such as OoD, and how semi-supervised learning can be performed to better identify unknowns. In addition, we review how to change the networks so they can be compared, and how we can use them for continual learning.

### Two-head network

**FIGURE 3:**
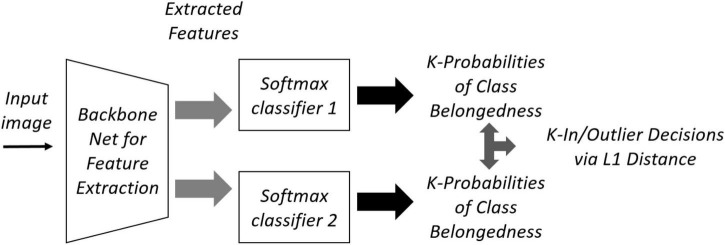
Two-head network for Out-of-Distribution (OoD) detection.


The two-head network uses two different randomly initialized softmax heads, *F_1* and *F_2*, that provide the same decision for labeled data, but different probability distribution for OoD data. Figure 3 shows the structure of a two-head network that shares a backbone. Originally there are two stages of training: pre-training with only labeled inlier data (ID), and fine-tuning with unlabeled OoD data. The training loss for labeled ID in the first stage is given by the cross-entropy:

(1) $$L_{c\,r\,o\,s\,s}^{t\,w\,o}\,\left(X\right)=-\frac{1}{X}\,\sum_{x_{b}\in X}\sum_{i=1}^{2}\text{log}\,\left(p_{i}\,\left(y_{b}|x_{b}\right)\right)$$

where {*x*_*b*_,*y*_*b*_} is the labeled ID samples, and index *i* is the head number.

In the second fine-tuning stage, the discrepancy loss is as follows:


(2) $$L_{d\,i\,s}^{t\,w\,o}\left(O\right)=max\{m-\frac{1}{\mu_{O}}\sum_{x_{o}\in \mu_{O}}d\left(p_{1}\left(y|x_{o}\right),p_{2}\left(y|x_{o}\right)\right\}$$

(3) $$d\left(p_{1}\left(y|x_{o}\right),p_{2}\left(y|x_{o}\right)\right)=\sum_{i=1}^{K}|p_{1}\left(y_{i}|x_{o}\right)-p_{2}\left(y_{i}|x_{o}\right)|$$

where *d*(⋅) is the *L*1 loss, and $O={\left\{x_{o}\right\}}_{o=1}^{\mu_{O}}$ is the set of unlabeled OoD data. In Eq. 2, *m* is a margin to prevent overfitting.

The OoD can be any irrelevant data to ID; it can be healthy leaves, fruit, runners or other images for strawberry disease recognition. Note that this OoD data is a type of bias that is inevitable in the OoD detector. Therefore, it is important to use them to increase the network’s performance. In Section “Experimental results of the two-head network,” we discuss the OoD data in more detail.

For continual learning, as displayed in Figure 1, the model can be retrained after performing an inference of unlabeled data. The inference process differentiates ID from OoD data. In the second-round training for continual learning, ID and OoD data are augmented by adding ID and OoD data.

### Semi-supervised OpenMatch

**FIGURE 4:**
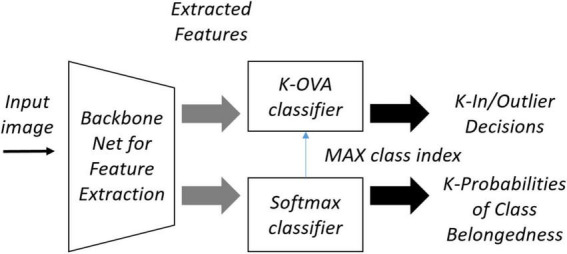
OpenMatch with softmax and One versus All (OVA) classifiers.


OpenMatch uses semi-supervised learning to improve OSR, where labeled and unlabeled data are mixed to create training data. Figure 4 shows the structure of the OpenMatch model. The base classifier consists of *K* one-vs-all (OVA) sub-classifiers ^*Dj*^ (⋅),*j* ∈ {1,…,*K*}, that share the feature extractor *F*(⋅), each of which determines whether it is an inlier or not with respect to the class. There is one more closed set classifier *C*(⋅), which gives the class label $\hat{y}$ in one of *K* classes for an input sample. The final unknown decision of whether it is an inlier or outlier is based on $D^{\hat{y}}\,\left(\cdot \right)$. The training of OpenMatch includes several losses and tries to minimize them. One of the losses is the cross-entropy loss for a closed set classifier:

(4) $$L_{c\,r\,o\,s\,s}\,\left(X\right)=-\frac{1}{B}\,\sum_{x_{b}\in X}y_{b}\,\text{log}\,\left(p\,\left(y|x_{b}\right)\right)$$

For a given batch of known data, $X={\left\{\left(x_{b},y_{b}\right)\right\}}_{b=1}^{B}$. In Eq. 4, *p*(*y*_*b*_|*x*_*b*_) is the probability of softmax output *y* for *x_b* from closed set classifier *C*(⋅). Another loss for the OVA outlier detection is defined as:


$$L_{O\,V\,A}\left(X\right)=\frac{1}{B}\sum_{b=1}^{B}-\log\left(p^{y_{b}}\left(t=0|x_{b}\right)\right)-min_{i\neq y_{b}}$$

(5) $$\log\left(p^{i}\left(t=1|x_{b}\right)\right)$$

where ^*pi*^ (*t* = 0|*x*_*b*_) and ^*pi*^ (*t* = 1|*x*_*b*_) represents the probabilities of *x_b* being an inlier or outlier for class *i*. For unlabeled data $U={\left\{\left(u_{b}\right)\right\}}_{b=1}^{\mu_{B}}$, there is another loss for OVA called entropy minimization, defined as:


$$L_{e\,m}\left(U\right)=-\frac{1}{\mu_{B}}\sum_{b=1}^{\mu_{B}}\sum_{j=1}^{k}p^{j}\left(t=0|u_{b}\right)\log\left(p^{j}\left(t=0|u_{b}\right)\right)$$

(6) $$+p^{j}\left(t=1|u_{b}\right)\log\left(p^{j}\left(t=1|u_{b}\right)\right)$$

Equation 7 is the soft open set consistency regularization (SOCR) loss for the OVA classifier to encourage the consistency of the output logits over any augmentation *A* to enhance the smoothness:


$$L_{O\,C}\left(U,A\right)=-\frac{1}{\mu_{B}}\sum_{b=1}^{\mu_{B}}\sum_{j=1}^{k}\sum_{t\in \left\{0,1\right\}}|p^{j}\left(t|A_{1}\left(u_{b}\right)\right)$$

(7) $$-p^{j}\left(t|A_{2}\left(u_{b}\right)\right)|$$

which emphasizes the consistency of OVA for differently augmented *A_1* and *A_2* unlabeled data.

During semi-supervised learning, unlabeled samples are taken as pseudo inliers to supplement the set of labeled data, if $p^{\hat{y}}\left(t=0|u_{b}\right)=\tau$, where $\hat{y}=a\,r\,g\,\max_{j}C\,\left(F\,\left(u_{b}\right)\right)$), after the training is stabilized.

The learning related to these pseudo inliers is called FixMatch (Sohn et al., 2020), and there is another corresponding loss to be minimized *L*_*fm*_. FixMatch is a combination of two approaches to semi-supervised learning: consistency regularization and pseudo-labeling (Sohn et al., 2020). Consistency regularization utilizes unlabeled data by relying on the assumption that the model should output similar predictions when fed perturbed versions of the same image (weak augmentation and strong augmentation). Pseudo-labeling leverages the idea of using the model itself to obtain artificial labels for unlabeled data. FixMatch progressively improves the performance of semi-supervised training (so-called curriculum learning) using pseudo-labeled data, where strong augmented pseudo inliers follow weak augmented ones. The FixMatch process can extend the decision boundary of known classes to allow the strongly augmented inliers to train models. Here, the corresponding loss can be described as:


(8) $$L_{f\,m}=-\sum_{b=1}^{\mu_{B}}𝕀\left(p^{\hat{y}}\left(t=0|u_{b}\right)>\tau \right)\text{log}p\left(\hat{y}|A\left(u_{b}\right)\right)$$

where 𝕀() is a set indicator function, and *A*(*u*_*b*_) stands for the strong augmented data for the pseudo inlier. Note that *L*_*fm*_ is the same as the cross-entropy losses except that they are calculated for pseudo inliers labeled by $\hat{y}$.

A contrastive loss can also be applied to OpenMatch to improve the accuracy and speed of the FixMatch training process (Sohn et al., 2020). FixMatch only considers consistency regularization between each high confidence pseudo inlier $\left(p^{\hat{y}}\left(t=0|u_{b}\right)>\tau \right)$ and its strong augmented version *A*(*u*_*b*_) by curriculum learning. On the other hand, contrastive regularization builds a pool of strong augmented samples of pseudo inliers where both positive and negative samples for pseudo-labeled data are included, and then tries to minimize the contrastive loss. In order to implement contrastive regularization, a pool of strong augmented unlabeled ID


Am(U)={u|′ub∈U,py^(t=0|ub)>τ,u=′iA(ub),

(9) 1≤i≤m}

is first built, in which the average contrastive loss is calculated using the positive and negative pairs. In Eq. 9, *m* strong augmented data for each pseudo inlier is included in *A*_*m*_(*U*). The contrastive loss for a sample *u*′ in *A*_*m*_(*U*) can be calculated by:


(10) $$r\,\left(u^{\prime }\right)=\frac{-1}{\left|\hat{P}\,\left(u^{\prime }\right)\right|}\,\sum_{p^{\prime }\in \hat{P}\,\left(u^{\prime }\right)}\log\frac{\text{exp}\,\left(\left\langle z_{u^{\prime }},z_{p^{\prime }}\right\rangle /T\right)}{\sum_{v^{\prime }\in A_{m}\,\left(U\right)/u^{\prime }}\text{exp}\,\left(\left\langle z_{u^{\prime }},z_{p^{\prime }}\right\rangle /T\right)}$$

where $\hat{P}\,\left(u^{\prime }\right)=\left\{p^{\prime }|p^{\prime }\in A_{m}\,\left(U\right)/u^{\prime },{\hat{q}}_{p^{\prime }}={\hat{q}}_{u^{\prime }}\right\}$ is a set of *p*′ which makes so-called pseudo positive pairs with *u*′, that has the same pseudo label ${\hat{q}}_{p^{\prime }}$ as ${\hat{q}}_{u^{\prime }}$. In Eq. 10, *T* is temperature scaling parameter, and *z*_*u’*_ is a normalized vector of the projection head.

**FIGURE 5:**
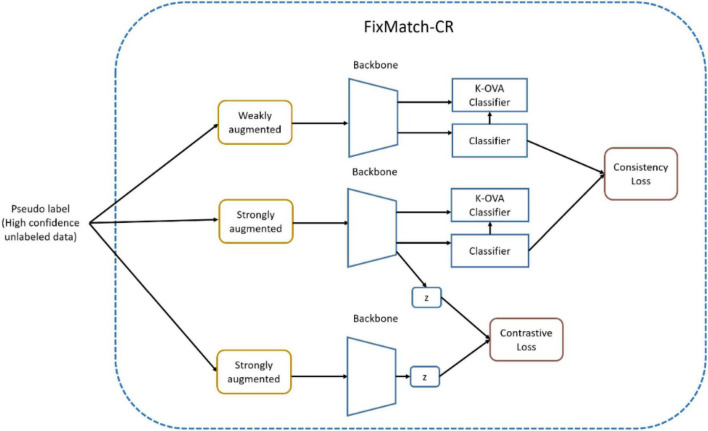
FixMatch with contrastive regularization in OpenMatch.

Figure 5 shows OpenMatch with FixMatch-included contrastive regularization. In semi-supervised training, the degree of confidence in ID or OoD data is determined by OVA classifiers and its pseudo label assigned by the softmax classifier, as demonstrated in Eq. 8. In Figure 5, FixMatch uses the pairs of weak and strong augmented pseudo inliers for consistency regularization of the softmax classifier, and the pool of strong augmented pairs of pseudo-inliers are utilized for the contrastive regularization of feature embedding as demonstrated in Eqs 9, 10.

### Discussions and comparison models

#### Outlier exposure

The two-head network explicitly includes OoD data with ID in its training for fine-tuning. On the contrary, OpenMatch improves OSR performance using semi-supervised learning, where the unlabeled data implicitly includes OoD data to better learn OVA according to the losses in Eqs 6, 7. OpenMatch assumes outlier exposure implicitly in unlabeled data.

As aforementioned, the set of OoD data prepared in the training process can be considered as a type of bias and a reason for overfitting, because it cannot include the large amount of OoD data; so-called unknown unknown space. Therefore, preparing an adequate and efficient set of OoD data for a specific domain is important. This is further discussed with the experimental results in the Section “Experimental results of the two-head network.”

#### Comparison models and continual learning

Two-stage training of the two-head network may be merged into single stage semi-supervised training, which starts with labeled and unlabeled data in the same manner as semi-supervised OpenMatch. In this case of two-head network, unlabeled ID can be treated as pseudo-labeled inliers after stabilizing the second stage of the fine-tuning process. Also, FixMatch with additional loss *L*_*fm*_ may be applied in the two-head network with contrastive regularization. However, this semi-supervised alignment of two-head network and OpenMatch is not intuitive. Therefore, we considered another modification to make a comparison between the two-head network and semi-supervised OpenMatch, as shown in Algorithm 1.

**Algorithm 1:** Modified two-head network.

Step 1: Train two classifiers with ID and perform fine-tuning with OoD data. Step 2: Inference unlabeled data including ID and OoD data. Step 3: Perform FixMatch with pseudo-labeled data in Step 2. Step 4: Perform fine-tuning with OoD data in Step 1 and Step 2.

**FIGURE 6:**
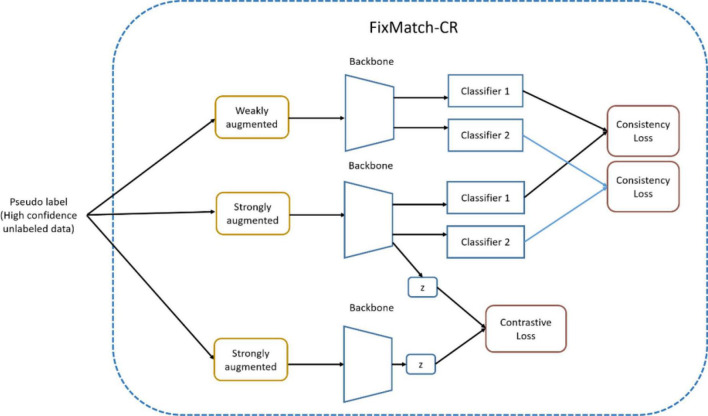
FixMatch with contrastive regularization in two-head network.

In Algorithm 1, the two-head network is retrained using FixMatch similar to OpenMatch in Step 3. FixMatch can be performed with pseudo-labeled data to improve the performance of the two-head classifier after the inference process of unlabeled data in Step 2, in the same manner as in the semi-supervised OpenMatch. Note that the high confidence pseudo inlier can be detected by Eq. 3, however, the discrepancy must be smaller than the threshold for unlabeled ID data. The set of ID samples with pseudo labels obtained from the softmax decision can then be used for FixMatch (with contrastive regularization), as shown in Figure 6. Each softmax classifier head is separately adjusted for consistency loss in FixMatch, and the backbone can learn contrastive loss.

Finally, the outliers in the inference process of Step 2 can be used to fine-tune the two classifier heads in Step 4. Instead of inherent inference and FixMatch in the training loop of semi-supervised OpenMatch, the two-head network performs FixMatch and fine-tuning after explicit inferencing of unlabeled data.

Also, in order to compare semi-supervised OpenMatch with the two-head network, OpenMatch can be disassembled into two stages; one training stage to learn the One versus All (OVA) and softmax classifiers similar to OoD detection, and a second stage to conduct semi-supervised learning of OpenMatch with labeled and unlabeled samples. In the second stage, the same OoD data used in the first stage is included in the outlier data. OSR does not intentionally include OoD data in the training phase, but the data was prepared for the two training stages of the disassembled OpenMatch. So, OpenMatch can also be treated as an OoD detector. Algorithm 2 summarizes the disassembled training process of OpenMatch. Note that the first training stage is prepared only for comparing the OoD detection capability with the two-head network.

**Algorithm 2:** Modified semi-supervised OpenMatch.

Step 1: Train softmax classifier and *k*-OVA classifiers with labeled ID and OoD data. Step 2: Perform semi-supervised training with labeled ID, unlabeled data, and OoD data from Step 1.

After the modifications, OpenMatch and the two-head network had approximately the same conditions for comparison, including the same data for training, inference, and retraining. Note that Steps 2 through Step 4 in Algorithm 1 are included in the semi-supervised training loop of OpenMatch, which closely aligns the two models.

As well, both Algorithm 1, 2 are associated with continual learning, because they include an inference process of unlabeled data, and the results are utilized to improve the performance of OSR or OoD detection. The two-head network explicitly improves the performance by adding Step 2s through Step 4 in Algorithm 2, while semi-supervised OpenMatch includes continual learning inherent in Step 2 of Algorithm 2.

The continual learning process that utilizes each of the two different models as a whole is possible as follows: When training a two-head network using Algorithm 1, it can be used to determine inliers and OoD data during the actual inference performed in Step 2. We can then use the classified unlabeled data with high confidence as pseudo labeled data for performing FixMatch in Step 3. The supplemented OoD data can then be continuously added to fine-tune the model in Step 3 to improve the performance. If it is necessary to cluster OoD data to obtain new labels, then the retraining from Step 1 is possible with the new head structure.

On the contrary, OpenMatch trained by Algorithm 2 can be used to recognize unknowns of OoD data and ID in the real inference process. As shown in Figure 1, an increased amount of labeled and unlabeled data, including confident ID and OoD data, can then be prepared for Step 2 in Algorithm 2. In this process, the unknowns of OoD data can be clustered to give new labels and include them for incremental learning. In this case, retraining from Step 1 is necessary to adjust the extended structure of the model.

#### Complexity of the two models

The complexity of the two-head network and OpenMatch is comparable, because the two-head network includes two softmax classifiers, while OpenMatch includes one softmax classifier and *k*-OVAs. If the number of class *K* is large, then the two-head network is simpler than OpenMatch; otherwise, OpenMatch is preferable in term of complexity.

## Experimental results

To conduct the experiment, we constructed a small dataset of strawberry diseases with unknowns that were used for training, validating the results, and testing. The experiments analyzed the effect of different types of OoD data, and the improvements of performance by adding technological components such as FixMatch with contrastive regularization.

### Dataset of strawberry diseases with unknowns

This paper considers a two-head network and OpenMatch classifiers for monitoring strawberry diseases. For validation purposes, we built a strawberry disease dataset which included eight disease categories: angular leafspot, anthracnose (fruit rot, runner), blossom blight, gray mold (fruit), leafspot, powdery mildew (fruit, leaf); as well as unknown diseases and/or disorders. Reportedly there are more than 70 strawberry diseases or disorders (Strawberry Diseases, 2022); however, only eight diseases are considered as known diseases in our work. Other diseases or disorders we do not consider in the experiment were treated as unknowns for continual learning. There are 13,089 images including 8,873 known diseases and 4,216 unknown diseases, as displayed in Table 2. Figure 2 shows prototypical images of known and unknown diseases. All the images were captured in more than 6 greenhouses by cellular phone cameras, because the system pursues a mobile application.

**TABLE 2:** Image dataset of strawberry diseases and unknown diseases.

Name of disease	Total no. of images	Training images	Validation images	Test images
Angular leafspot (ALS)	818	498	184	136
Anthracnose fruit rot (AFR)	188	137	32	19
Anthracnose runner (AR)	232	129	33	70
Blossom blight (BB)	1,898	1,410	264	224
Gray mold (GM)	1,303	1,003	171	129
Leaf spot (LS)	2,299	1,703	360	236
Powdery mildew fruit (PML)	397	236	77	84
Powdery mildew leaf (PML)	1,738	1,257	232	249
Unknown or OoD diseases or disorders	4,216	1,346	1,435	1,435
Total	13,089	7,719	2,788	2,582

### Experimental results of the two-head network

#### Training the two-head network for comparison with OpenMatch

The training of the two-head network consisted of two stages: the pre-training of each head of softmax classifiers, and fine-tuning with OoD data. To compare the two-head network with semi-supervised OpenMatch, and to show the applicability of continual learning, we added several steps in the training of the two-head network, as shown in Algorithm 2.

Step 1 trained the two heads of softmax classifiers with labeled ID and performed fine-tuning using OoD data to maximize the discrepancy between the decisions in the two heads. As explained in Section “Materials and methods” Step 2 performed the inferencing of unlabeled data in the same manner as in semi-supervised training of OpenMatch. After the inference, the pseudo inliers or outliers were obtained from the trained two-head network. The high confidence labeled pseudo inliers were then used by FixMatch with contrastive regularization, as displayed in Figure 6. Finally, Step 4 performed fine-tuning with the original OoD data one additional time.

Algorithm 1 used the same labeled and OoD data for training, inferencing to find the pseudo inliers, and fine-tuning, similarly to the disassembled OpenMatch in Algorithm 2, in order to compare the two different models.

To train model, the original dataset in the second column of Table 2 was divided into training, validation, and test data. The training was performed using a random online selection of (weak) augmented data, visually rotated at 90, 180, and 270 degrees. For the intermediate inference stage, we used 2,659 unlabeled inliers and OoD data.

**FIGURE 7:**
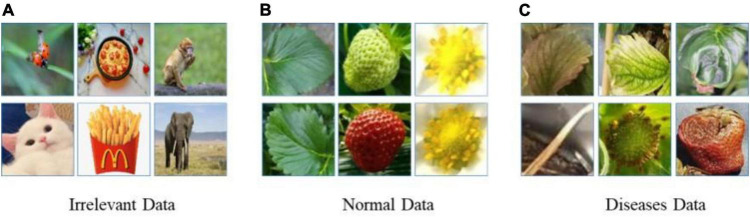
Three types of Out-of-Distribution (OoD) data.

As previously discussed, in the fine-tuning stage in Step 1 of Algorithm 1, there were several possible ways to build OoD data, because it could draw from a large unknown data space. One way was to include only irrelevant data randomly selected from the ImageNet dataset, such as bugs, food, and trees. Another method was to include normal (healthy) strawberry data such as flowers, leaves, runners, and fruit. In addition, we could include unknown diseases or disorders that were not part of the known classes. We prepared the same amount of three types of OoD data: irrelevant data, healthy strawberry data, and unknown suspected disease data. Figure 7 shows the different types of OoD data samples used to train the models. The effects of the three different types of OoD data on the performance of the models is compared in Table 3 and discussed in Section “Experimental results and discussion.”

**TABLE 3:** Performance of the two-head network with different experimental settings.

1st						2nd	Improved 2nd	
Irrelevant	Normal	Unknowns (diseases/disorders)	I + N	N + D	I + N + D	Using pseudo label	Without contrastive regularization	With contrastive regularization
**Accuracy**								
0.785	0.855	0.865	0.858	0.862	0.861	0.883	0.901	0.924
**AUROC**								
0.828	0.917	0.919	0.912	0.926	0.923	0.940	0.951	0.972

1st: 1st round training followed by fine-tuning. 2nd: 2nd round without FixMatch followed by fine-tuning. Improved 2nd: 2nd round FixMatch-CR followed by fine-tuning. I + N: Fine-tuning OoD data is a mix of irrelevant and normal data. N + D: Fine-tuning OoD data is a mix of normal and diseases data. I + N + D: Fine-tuning OoD data is a mix of irrelevant, normal, and diseases data.

For FixMatch, we required sets of weak and strong augmentation to gradually improve classification performance. In the experiment, the geometrically transformed images, as previously mentioned, were used for weak augmentation. For strong augmentation, images with color and brightness changes and different degrees of rotations were included, and an augmentation was randomly chosen among 36 different alternatives during FixMatch training.

**FIGURE 8:**
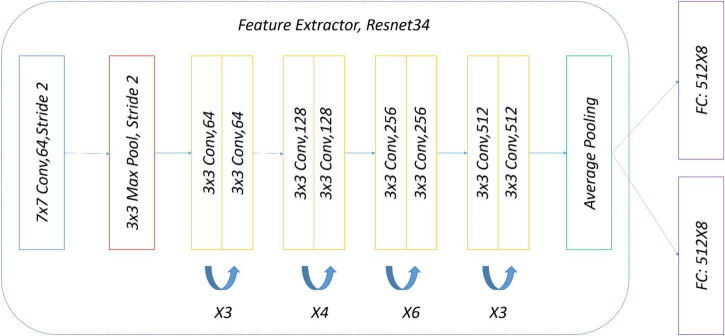
Two-head network for experiment.

The precise structure of the two-head network used in the subject experiment is shown in Figure 8. ImageNet pre-trained by ResNet34 was selected as a backbone for simplicity, and there were two eight-way softmax classifiers.

To train the two-head softmax classifiers, an SGD optimizer was selected with a learning rate that decayed from 0.01. For fine-tuning, the fixed learning rate was set to 2=10^−4^ using the same SGD optimizer. The batch size was 64 and the number of epochs for pre-training and fine-tuning were 300 and 10, respectively.

#### Experimental results and discussion

We used classification accuracy with unknown disease and AUROC as evaluation metrics. TP, TN, FP, FN are used to denote true positives, true negatives, false positives, and false negatives, respectively. Accuracy is the ratio of correctly classified samples (TP + TN) to the total number of samples. AUROC is the Area Under the Receiver Operating Characteristic curve and can be calculated by the area under the FPR (FPR = FP/(FP + TN)) against the TPR (TPR = TP/(TP + FN)) curve. We also used precision and recall in the confusion matrix. Precision refers to the proportion of the true positive class (TP) among all judged positive classes (TP + FP). Recall refers to the proportion of all true positive classes (TP + FN) that are judged as positive classes (TP).

Table 3 shows the performance of the pre-training and fine-tuning of the two-head network. In Table 3 we compare performance from the different types of OoD data with the same labeled inlier training data. The combined OoD data, using normal (healthy) strawberry parts including leaves, flowers, fruit, and runners, as well as unknown diseases (or disorders), resulted in satisfactory performance recognizing diseases as well as unknowns. Note that irrelevant OoD data was not helpful to train the two-head network, even though it was included in the mixed OoD data of normal and unknowns, displayed in the sixth column of Table 3. The results in Table 3 show that OoD data selected from healthy plant parts can be helpful, which is useful for practical applications of plant disease monitoring.

Note that the selection of OoD samples is a bias in OoD detection. Biased will inevitably be introduced if the successful performance of the model requires outlier exposure. Generalized ODIN, a similar OoD detector without the bias, demonstrated 81.9% accuracy and 0.894 of AUROC using the same strawberry data. The set of OoD samples, composed of healthy parts and unknowns, might be an inevitable but reasonable bias to enhance the performance of fine-grained unknown disease detection in plants.

**FIGURE 9:**
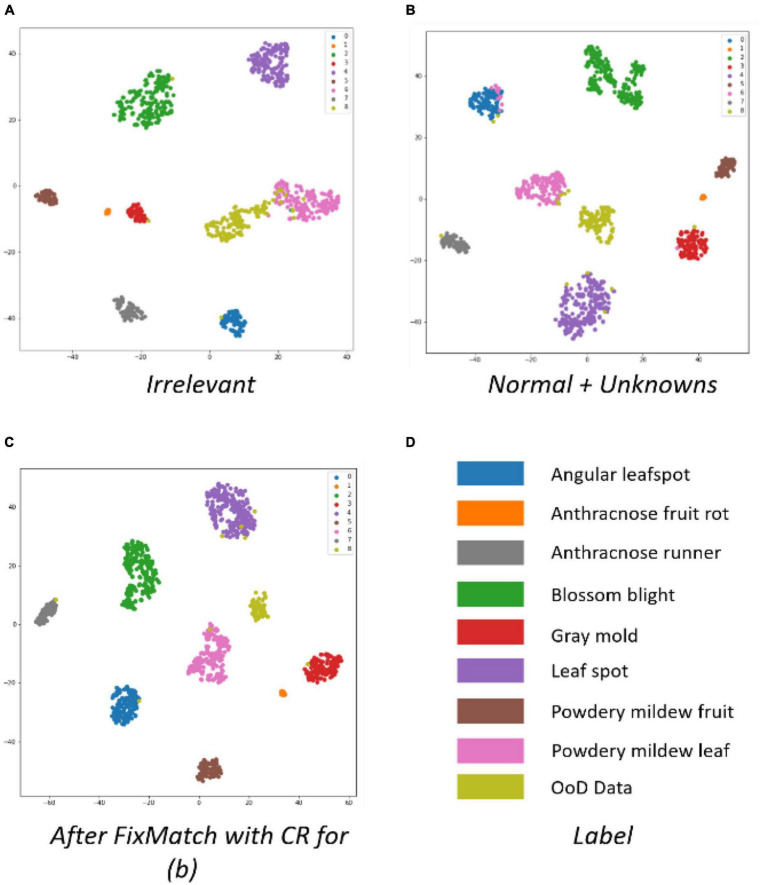
Comparison of cluster structures using t-SNE.

Figure 9 shows t-SNE images taken after different kinds of OoD data was trained. Figure 9B shows a more compacted cluster structure of different classes than Figure 9A, which correspond to the first and fifth columns of Table 3, respectively. The t-SNE images demonstrate why irrelevant outliers are not helpful in judging OoD, even if they are exposed during the training of an OoD detector. The irrelevant OoD samples cannot be used as hard negative samples to help the ID class become compact. Therefore, we built the OoD data using normal healthy parts and unknown diseases (or disorders) of strawberries for the rest of experiments.

In the inference stage, we prepare 2,659 images of inliers and OoD data, and select 1,350 high-confidence inliers with pseudo-labels. In order to find the required confident ID, we used two thresholds: the threshold of *L1* distance in Eq. 3, and the maximum class probability of two softmax classifiers. The former threshold was determined using a grid search on (0,1) to identify the maximum detection accuracy of OoD in the fine-tuning stage of Step 1, and the latter was 0.95; the same value as in Yu and Aizawa (2019).

The pseudo inliers were used to perform FixMatch with contrastive regularization in order to upgrade the performance of the two closed-set classifiers. Thereafter, the two-head network was fine-tuned with supplemented OoD data determined in the inference stage. The final result of the fine-tuning is shown in the last two columns of Table 3. Note that there was approximately a 3.6 (5.9) % gain using FixMatch (with contrastive regularization) and fine-tuning. When pseudo-ID obtained from the inference stage was used to train the two-head classifier without FixMatch of Step 3 in Algorithm 1, the performance decreased, as displayed in the seventh column of Table 3.

The t-SNE in Figure 9 shows that FixMatch with contrast regularization can make the intra-class distance more compact and the inter-class distance larger.

Note that this sequence of inferencing unknown data, using FixMatch with pseudo inliers, and fine-tuning with outliers, can be repeated to continuously improve the performance of the network.

Table 4 shows a confusion matrix after FixMatch with contrastive regularization followed by fine-tuning. Note that the class label was given only when the decisions from the two heads were consistent. Otherwise, the input image was treated as unknown. Leaf diseases like angular leafspots and powdery mildew (leaf) had reduced recall due to confusion with unknowns. Furthermore, the leaf diseases of angular leafspots, leaf spot, and gray mold (fruit) were inaccurately identified due to confusion with unknowns. The unknown detection results included 94.9% recall and 91.9% precision.

**TABLE 4:** Confusion matrix of the final two-head network (Fixmatch-CR).

	ALS	AFR	AR	BB	GM	LS	PWF	PML	OoD	Recall
Angular leaf spot (ALS)	122	0	0	0	0	0	0	0	14	0.897
Anthracnose fruit rot (AFR)	0	17	0	0	0	0	0	0	2	0.895
Anthracnose runner (AR)	0	0	68	0	0	0	0	0	2	0.971
Blossom blight (BB)	0	0	0	222	0	0	0	0	2	0.991
Gray mold (GM)	0	0	0	0	120	0	0	0	9	0.930
Leaf spot (LS)	0	0	0	0	0	235	0	0	1	0.996
Powdery mildew fruit (PMF)	0	0	0	0	0	0	76	0	8	0.905
Powdery mildew leaf (PML)	1	0	0	0	0	0	0	166	82	0.667
Unknowns (OoD)	8	0	1	0	10	24	0	30	1,362	0.949
Precision	0.931	1.000	0.986	1.000	0.923	0.907	1.000	0.847	0.919	

**FIGURE 10:**
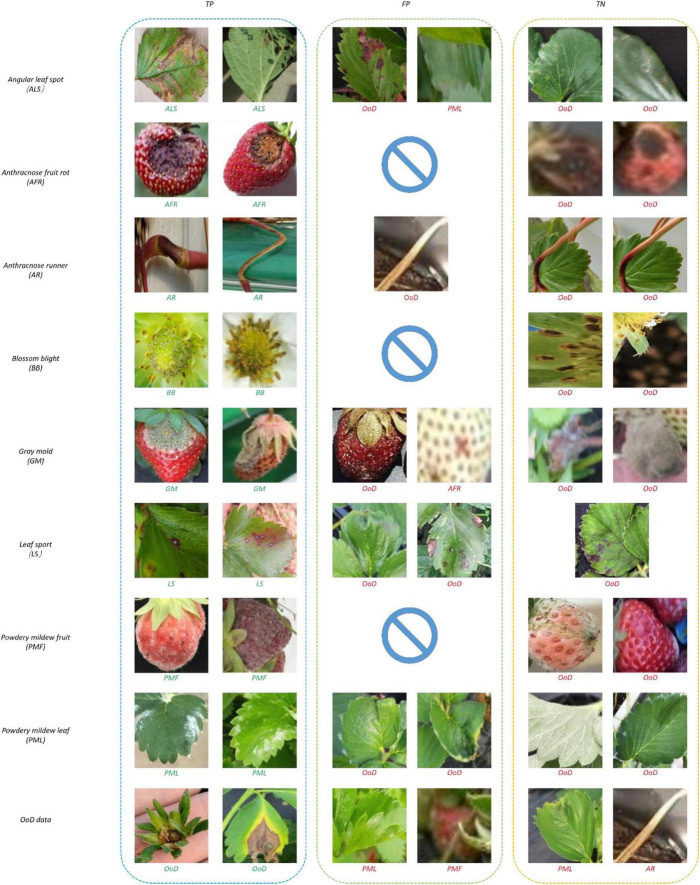
Recognition results of correct and incorrect classification.

Figure 10 shows samples of recognition results. In Figure 10, all the true negatives (TNs) of leaf and fruit diseases were categorized as unknowns. Note that there were many false positives (FPs) and TNs due to image quality problems including bad illumination and blurring. In addition, some diseases featured small-sized symptoms which were difficult to discern and hard to differentiate, even by human eyes. There were no FPs of flower or runner diseases, due to their distinct shape compared to leaf or fruit diseases.

### Experimental results of OpenMatch

#### Training of OpenMatch for comparison with the two-head network

We dissembled the end-to-end semi-supervised learning into Algorithm 2 in order to compare OpenMatch with the two-head network, as described in Section “Discussions and comparison models.” In the first stage, OpenMatch was trained with the same labeled and unlabeled OoD data, similar to the first stage of the two-head network. The OpenMatch was initially treated as if it was an OoD detector. We then performed the semi-supervised OpenMatch training which included inferencing unlabeled data to find the pseudo inliers, as well as using FixMatch with contrastive regularization.

**FIGURE 11:**
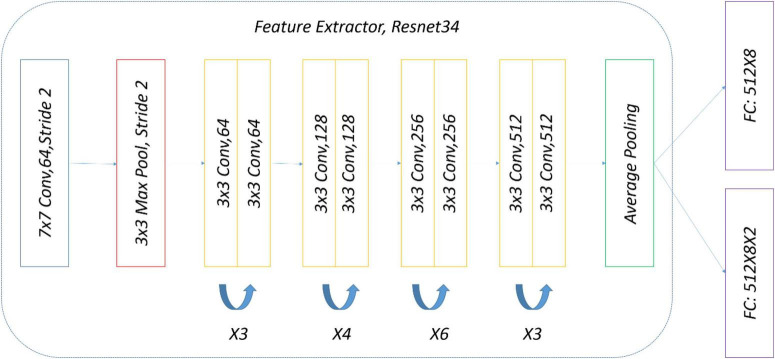
Experimental OpenMatch design.

To ensure a fair comparison with the two-head network, the same data and the same weak (strong) augmentation methods at each training stage were used. The precise structure of OpenMatch used in the experiment is shown in Figure 11; ImageNet pre-trained ResNet34 was selected again as a DNN backbone, and there was an eight-way softmax closed-set classifier and 8 OVA classifiers, due to the identification of eight strawberry diseases.

#### Experimental results and discussion

To train OpenMatch classifiers, an SGD optimizer was selected with a learning rate that decayed from 0.01. For fine-tuning, the fixed learning rate was set to 2=10^−4^ using the same SGD optimizer. The batch size was 64 and the number of epochs for pre-training and fine-tuning were 300 and 10, respectively.

Table 5 shows the performance of the disassembled OpenMatch across different experimental settings. As a dataset of healthy parts and unknown diseases was identified as the most helpful OoD data, only those samples were used in order to simplify the experiment.

**TABLE 5:** Performance of disassembled OpenMatch with different experimental settings.

	OpenMatch as OoD detector	OpenMatch without FixMatch	Semi-supervised training with FixMatch	
		Using pseudo label	Without CR	With CR
Accuracy	0.865	0.888	0.900	0.922
AUROC	0.928	0.944	0.951	0.971

In the second semi-supervised training stage of OpenMatch, the same 2,659 images of inlier and OoD data used in the inference stage of the two-head network were prepared. During second stage training, the high confidence pseudo inliers were detected and applied to perform FixMatch with contrastive regularization, in order to upgrade the performance of the disassembled OpenMatch classifiers. In the experiment, the threshold τ in Eq. 8 was 0.95 to detect pseudo inliers. The result of the training is shown in Table 5.

Using OpenMatch without the semi-supervised method as the OoD detector yields an accuracy of 86.5%, as shown in the first column of Table 5. Note that only the OoD samples were fed as unlabeled data, similar to the fine-tuning stage of the two-head network. The performance of OpenMatch as an OoD detector was comparable with the 86.2% accuracy of the two-head network.

Usually, OSR does not make use of unknowns in the training phase, so that there is no bias regarding the type of unknowns. While OpenMatch with OoD data samples was biased due to unknown exposure during training, it was an inevitable but reasonable bias, similarly observed in the two-head network. When we applied OpenMax, a well-known OSR technique, the accuracy and AUROC were 70.1% and 0.812, respectively. The outlier exposure provided a significant 16.4% increase in accuracy, even though the OpenMax and OpenMatch structures were different.

By combining the semi-supervised training of OpenMatch with FixMatch (with contrastive regularization), accuracy improved as much as 3.5 (5.7) %, as displayed in Table 5, which was comparable with the accuracy of the retrained two-head network. The accuracy improvement might have been a result of semi-supervised learning with unlabeled inliers and outliers. The semi-supervised setting without FixMatch, where the high confident pseudo inliers were included in the semi-supervised OpenMatch, provided a small 2.3% gain in accuracy, as shown in the third column of Table 5. It can be seen from Table 5 that adding the contrast regularization technique can effectively improve the performance of FixMatch.

**FIGURE 12:**
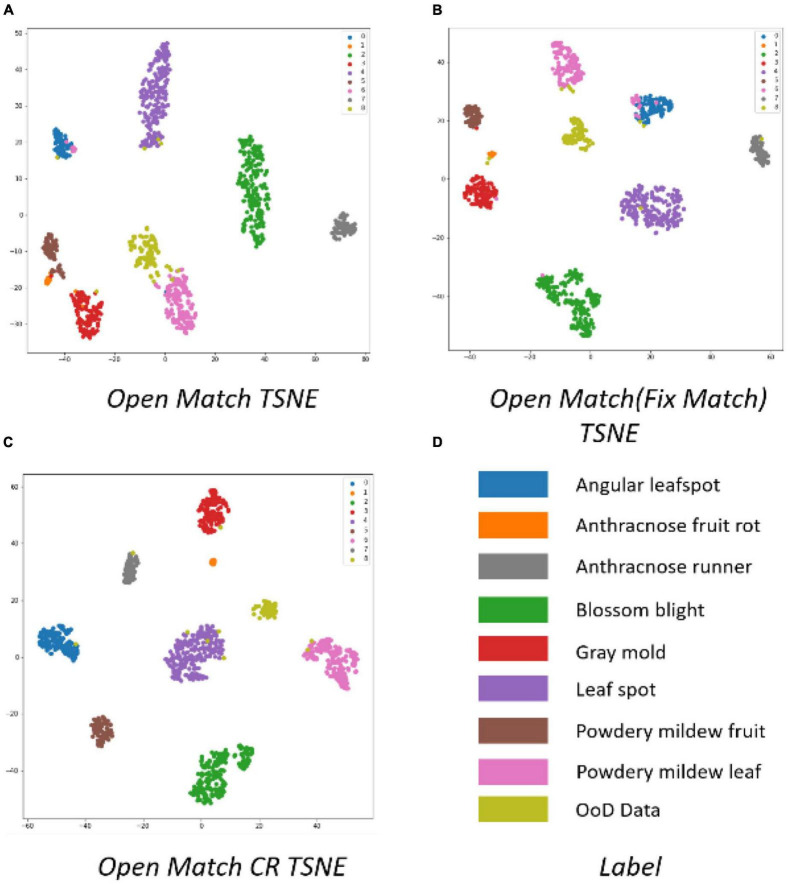
Comparison of cluster structures of disassembled OpenMatch using t-SNE.

Figure 12 shows t-SNE images after OpenMatch training. The more compact cluster structure of classes was a result of the semi-supervised learning of OpenMatch and contrastive regularization in Figures 12B,C, respectively.

Table 6 shows the confusion matrix for the best experimental performance, which featured semi-supervised OpenMatch with contrastive regularization. The unknown detection results included 95.5% recall and 91.3% precision, which was comparable with the two-head network. Similar to the results of the two-head network, leaf diseases such as angular leaf spots, powdery mildew, and gray mold fruit were confused with unknowns.

**TABLE 6:** Confusion matrix of the retrained OpenMatch-CR.

	ALS	AFR	AR	BB	GM	LS	PWF	PML	OoD	Recall
Angular leaf spot (ALS)	121	0	0	0	0	0	0	0	15	0.890
Anthracnose fruit rot (AFR)	0	16	0	0	0	0	0	0	3	0.842
Anthracnose runner (AR)	0	0	69	0	0	0	0	0	1	0.986
Blossom blight (BB)	0	0	0	222	0	0	0	0	2	0.991
Gray mold (GM)	0	0	0	0	119	0	0	0	10	0.922
Leaf spot (LS)	0	0	0	0	0	235	0	0	1	0.996
Powdery mildew fruit (PMF)	0	0	0	0	0	0	77	0	7	0.917
Powdery mildew leaf (PML)	5	0	0	0	0	0	0	153	91	0.614
Unknowns (OoD)	3	0	1	0	5	14	0	41	1,371	0.955
Precision	0.831	0.950	0.986	0.991	0.899	0.946	0.974	0.931	0.898	

**FIGURE 13:**
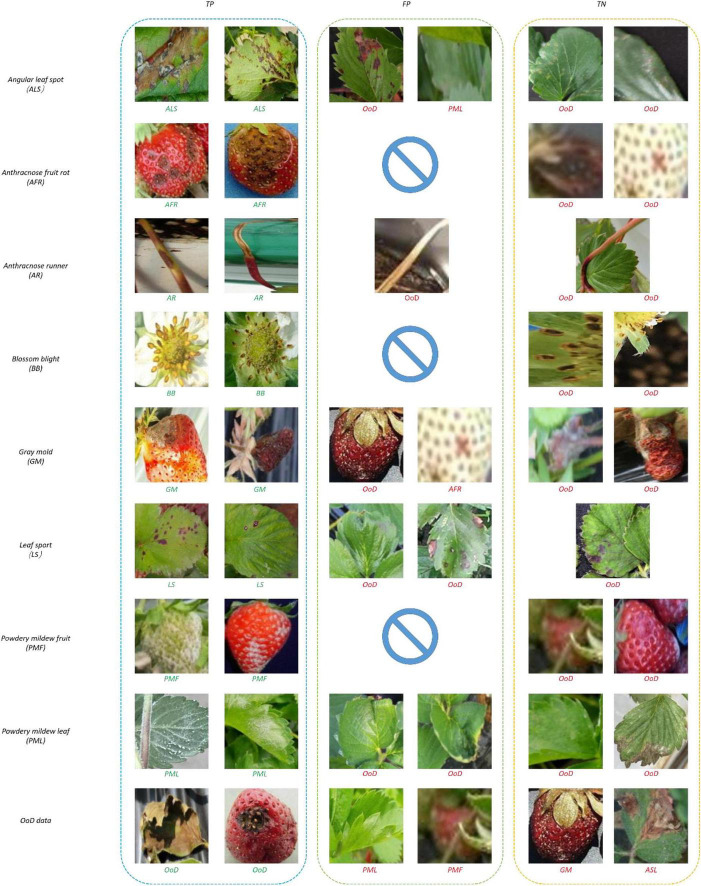
Recognition results of correct and incorrect classification.

Figure 13 shows samples of recognition results. In Figure 13, all the TNs of leaf and fruit diseases were categorized as unknowns, similar to the results of the two-head network. Image quality was the primary reason for misclassification of FPs and TNs, as seen in Figure 13.

## Conclusion

For continuous learning in the plant disease identification process, an unknown disease or condition should first be distinguished from a known disease. This paper examined with two different but related deep learning-based techniques the detection of unknown plant diseases, including OSR and OoD detection. We chose the two-head network using OoD detection and semi-supervised OpenMatch using OSR technology, which explicitly and implicitly assume outlier exposure, respectively.

We carefully review the two models, and performed modifications in order to compare their performance classifying known diseases as well as detection of unknown diseases. For the experiment, we built an image dataset of eight strawberry diseases. Experiments on the dataset show that assuming outlier exposure during training is helpful for detecting unknown diseases. The experimental results also demonstrated that a careful selection of OoD samples for training is important to achieve better performance. Additionally, we demonstrated that FixMatch in semi-supervised OpenMatch can be successfully added into a two-head network, with contrastive regularization, to improve performance. Both OoD detection and OSR provided reasonable and comparable performance, as they were more than 92% accurate classifying the eight strawberry diseases and detecting unknown diseases. We believe the methods used in our experiment are general in nature, allowing them to be effectively applied to any type of plant disease monitoring.

## Data availability statement

The raw data supporting the conclusions of this article will be made available by the authors, without undue reservation.

## Author contributions

KJ and JL: conceptualization and writing—original draft preparation. KJ, JY, and JL: methodology. KJ, JY, and U-OD: formal analysis and investigation. KJ, JL, and HK: writing—review and editing. JL and HK: resources. JL: supervision. All authors contributed to manuscript revision, read, and approved the submitted version.
