# Supplementary figures and images for: Detection of unknown strawberry diseases based on OpenMatch and two-head network for continual learning
Source: Front Plant Sci. 2022 Sep 15;13:989086. doi: 10.3389/fpls.2022.989086 (PMC9520169; doi:10.3389/fpls.2022.989086)

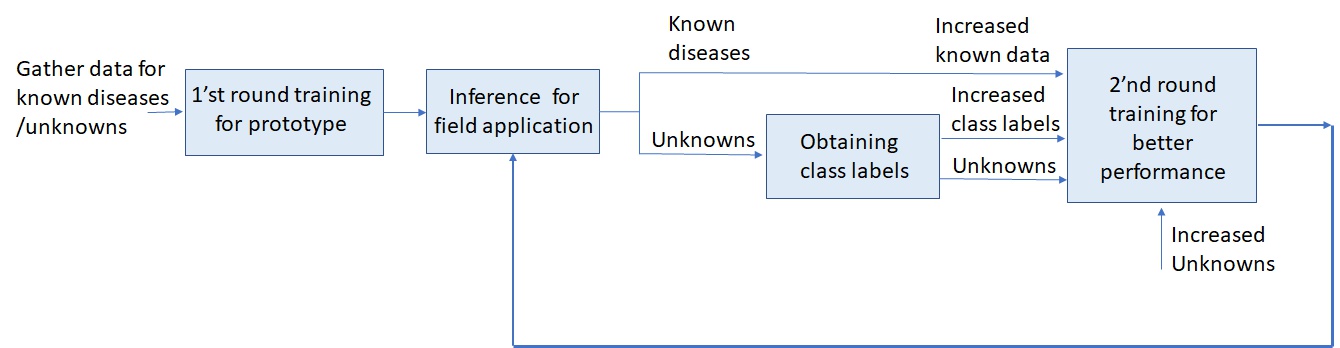

Supplement: Supplementary file 1 [file Data_Sheet_1.ZIP › fig1.jpg]

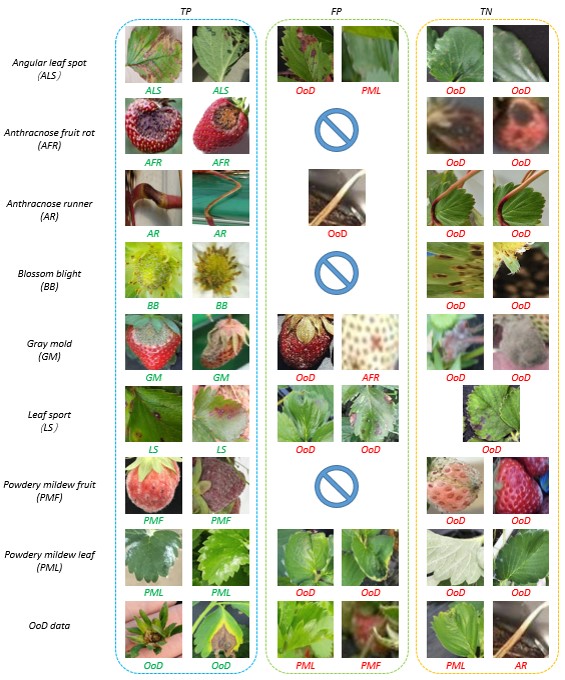

Supplement: Supplementary file 1 [file Data_Sheet_1.ZIP › fig10.jpg]

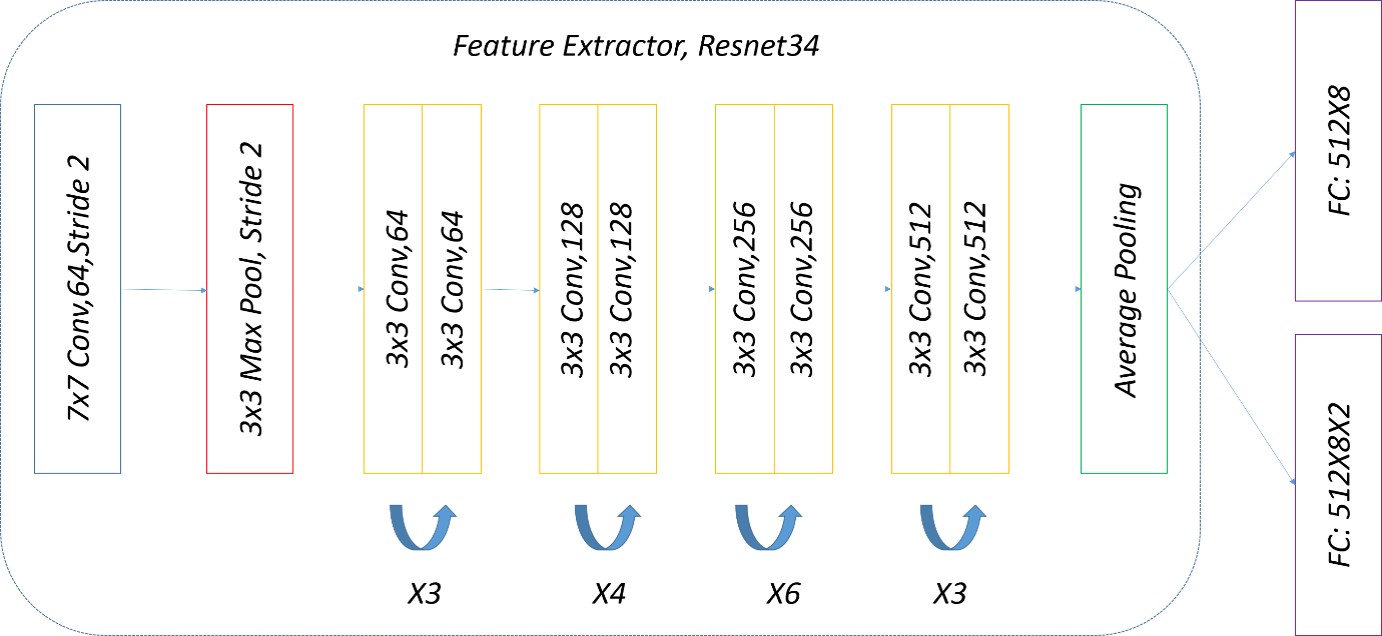

Supplement: Supplementary file 1 [file Data_Sheet_1.ZIP › fig11.jpg]

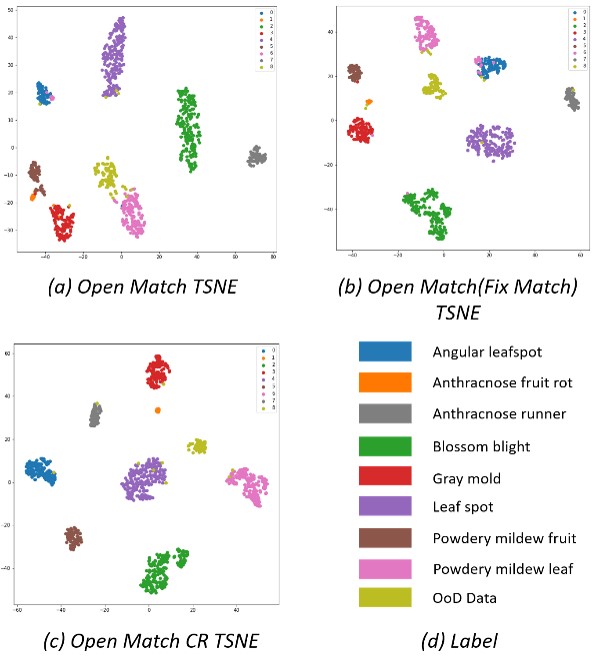

Supplement: Supplementary file 1 [file Data_Sheet_1.ZIP › fig12.jpg]

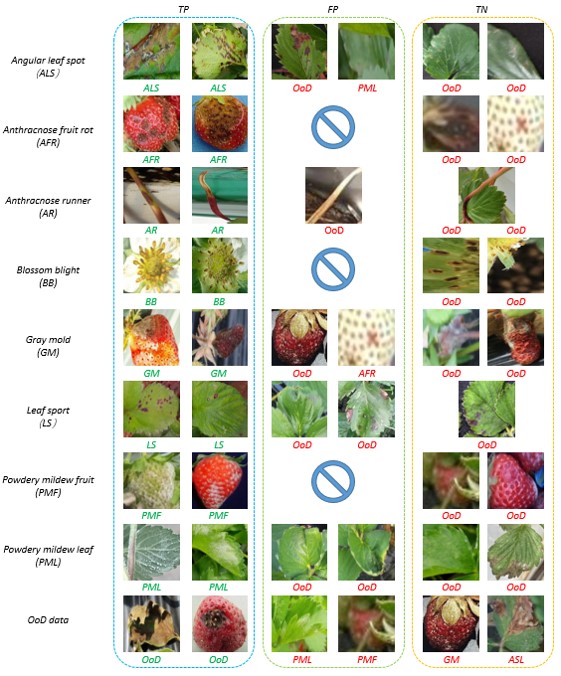

Supplement: Supplementary file 1 [file Data_Sheet_1.ZIP › fig13.jpg]

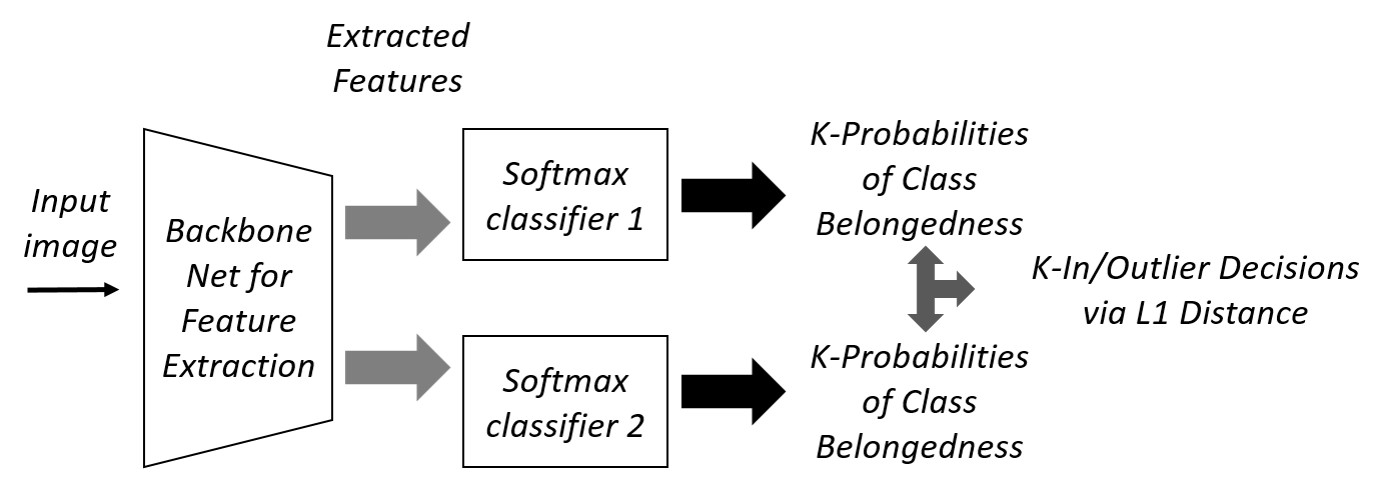

Supplement: Supplementary file 1 [file Data_Sheet_1.ZIP › fig2.jpg]

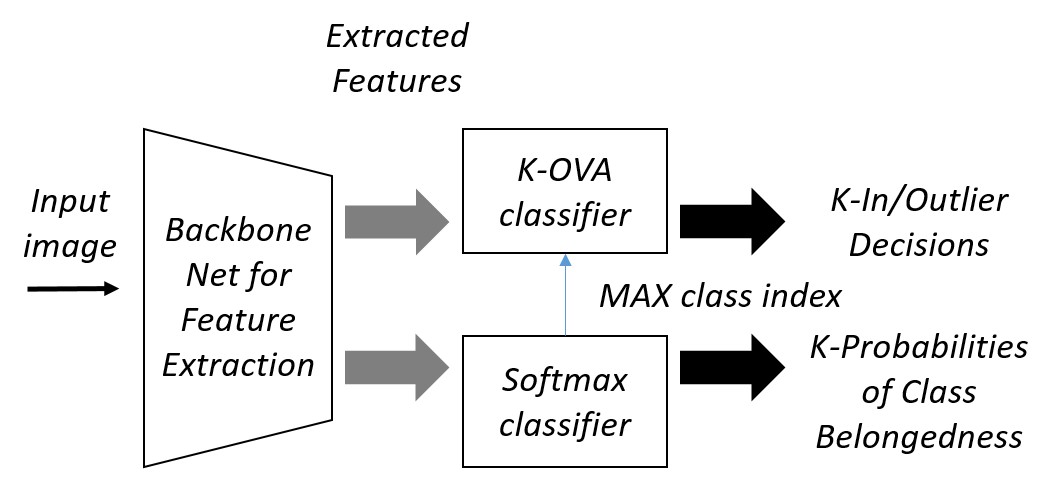

Supplement: Supplementary file 1 [file Data_Sheet_1.ZIP › fig3.jpg]

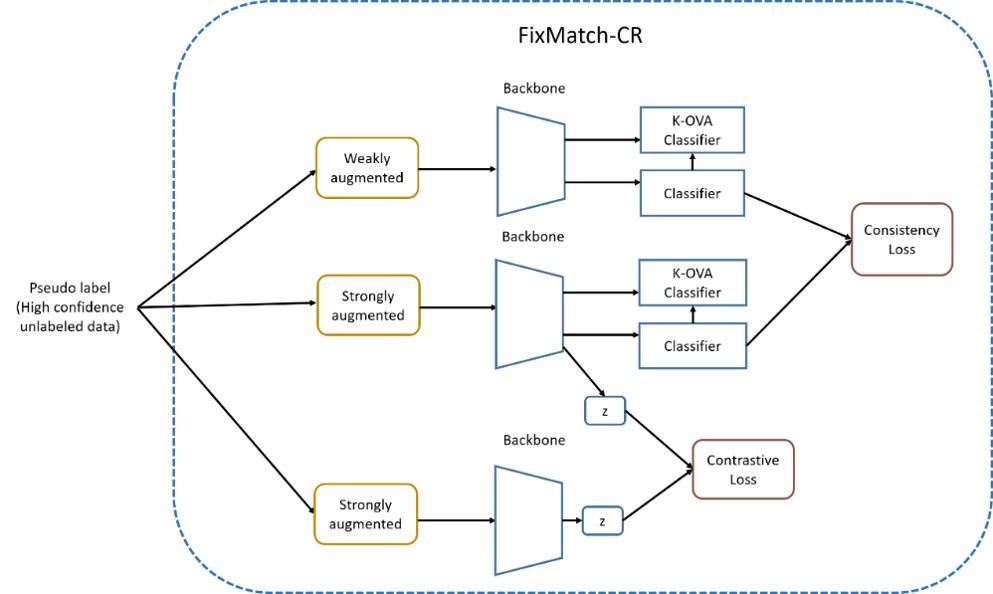

Supplement: Supplementary file 1 [file Data_Sheet_1.ZIP › fig4.jpg]

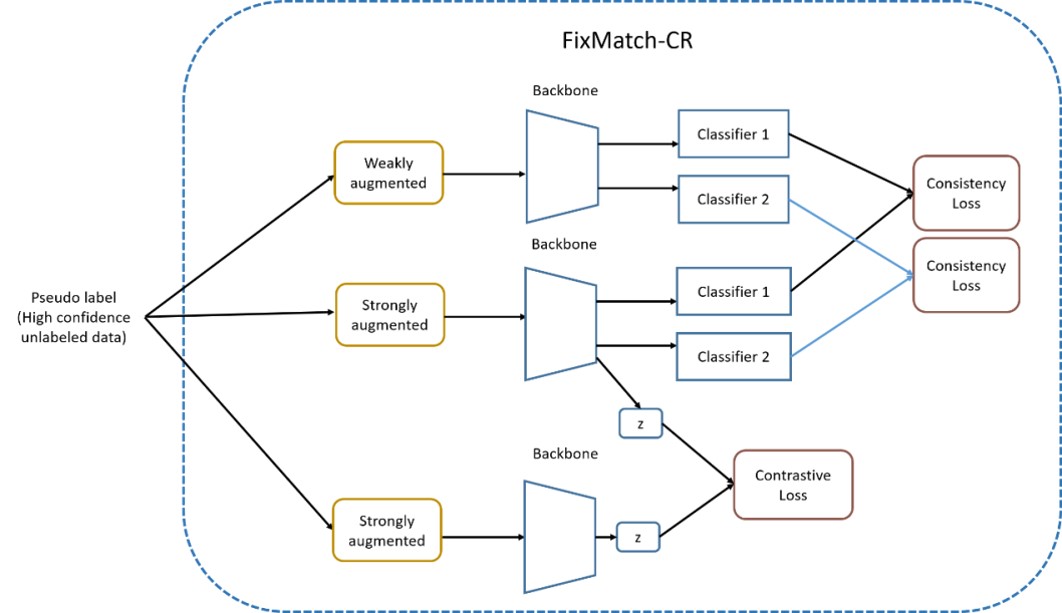

Supplement: Supplementary file 1 [file Data_Sheet_1.ZIP › fig5.jpg]

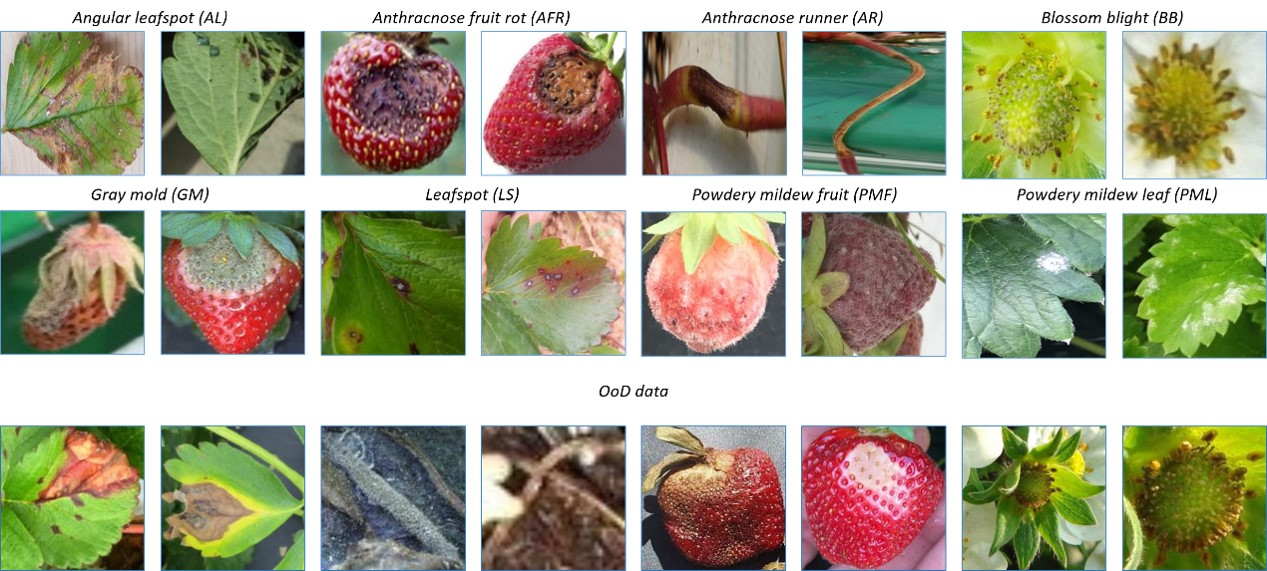

Supplement: Supplementary file 1 [file Data_Sheet_1.ZIP › fig6.jpg]

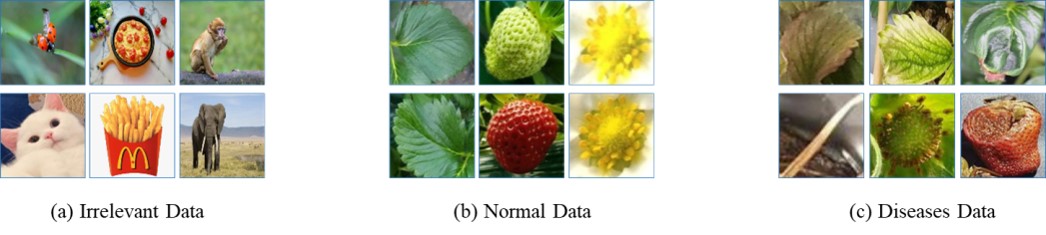

Supplement: Supplementary file 1 [file Data_Sheet_1.ZIP › fig7.jpg]

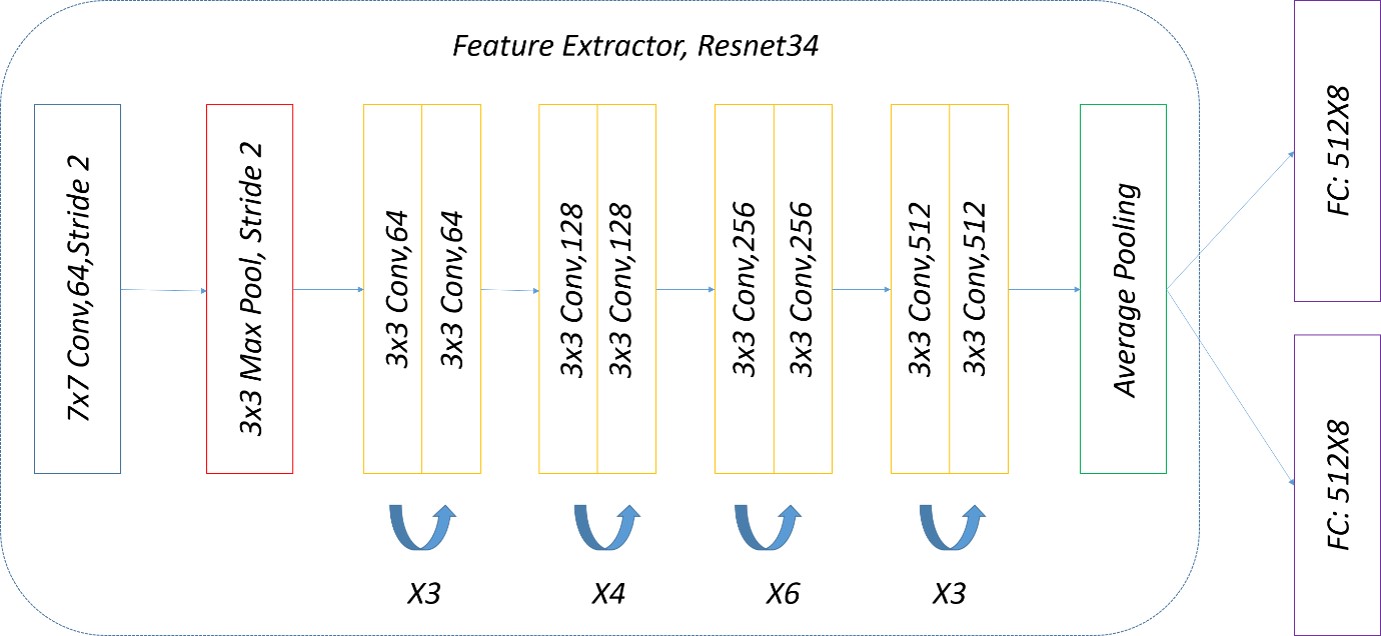

Supplement: Supplementary file 1 [file Data_Sheet_1.ZIP › fig8.jpg]

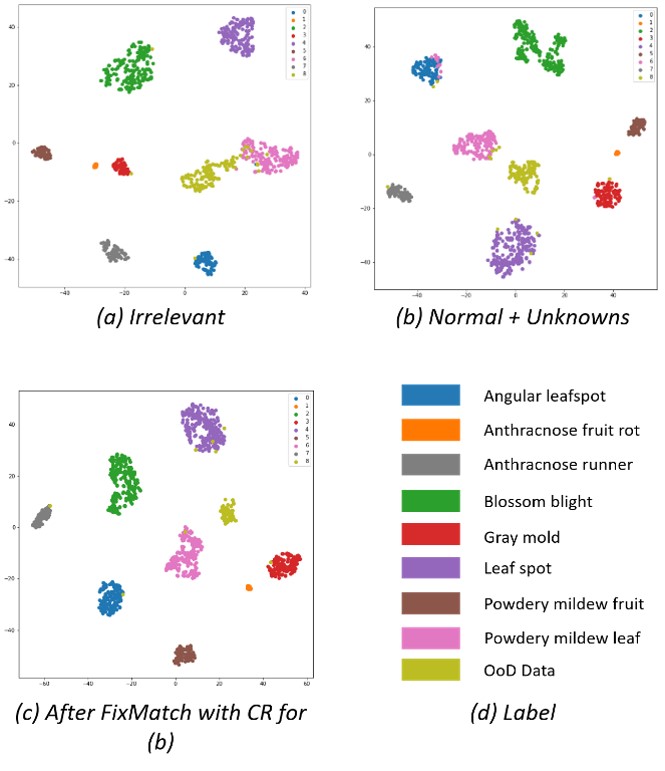

Supplement: Supplementary file 1 [file Data_Sheet_1.ZIP › fig9.jpg]

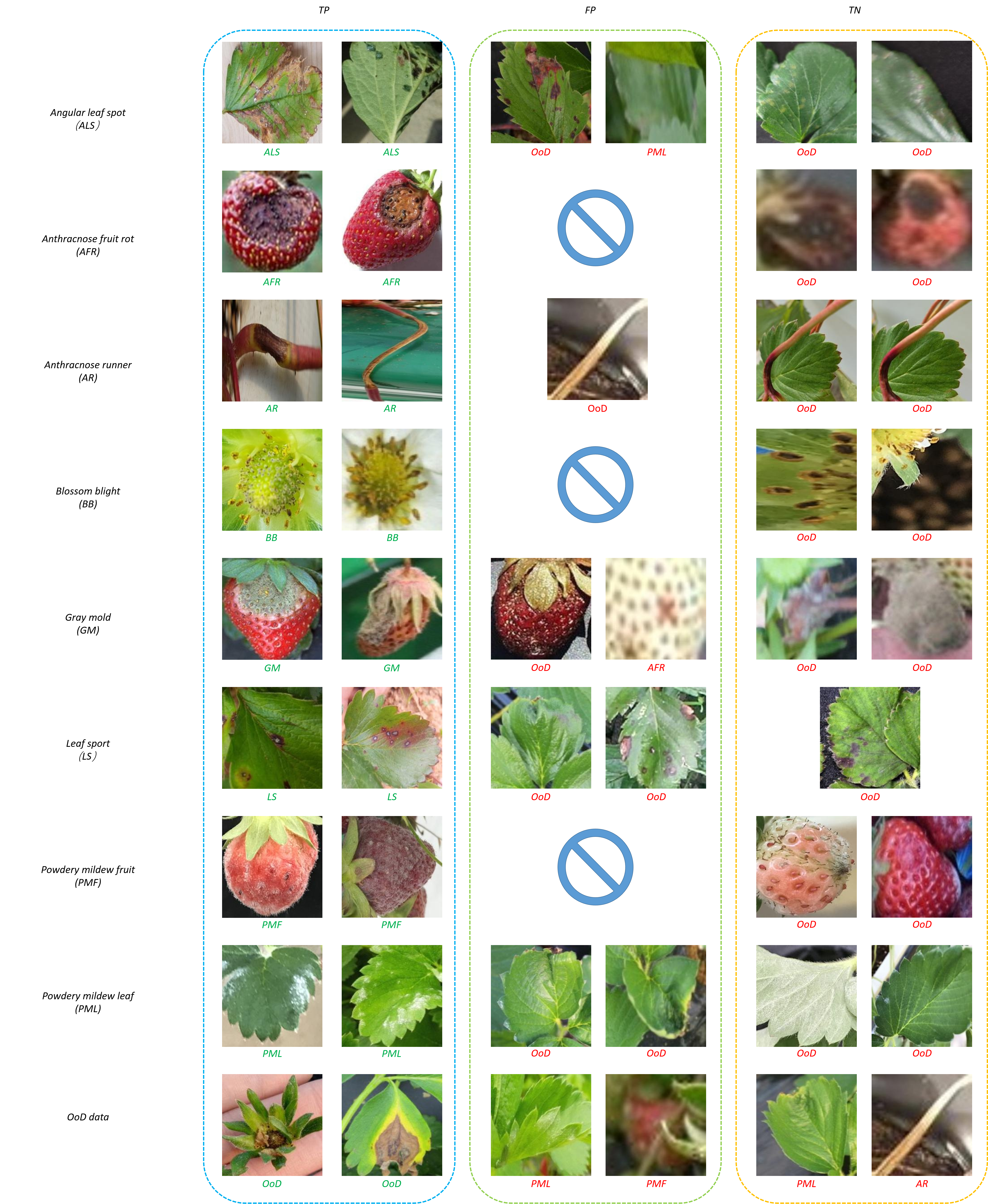

Supplement: Supplementary file 2 [file Data_Sheet_2.ZIP › fig10.png]

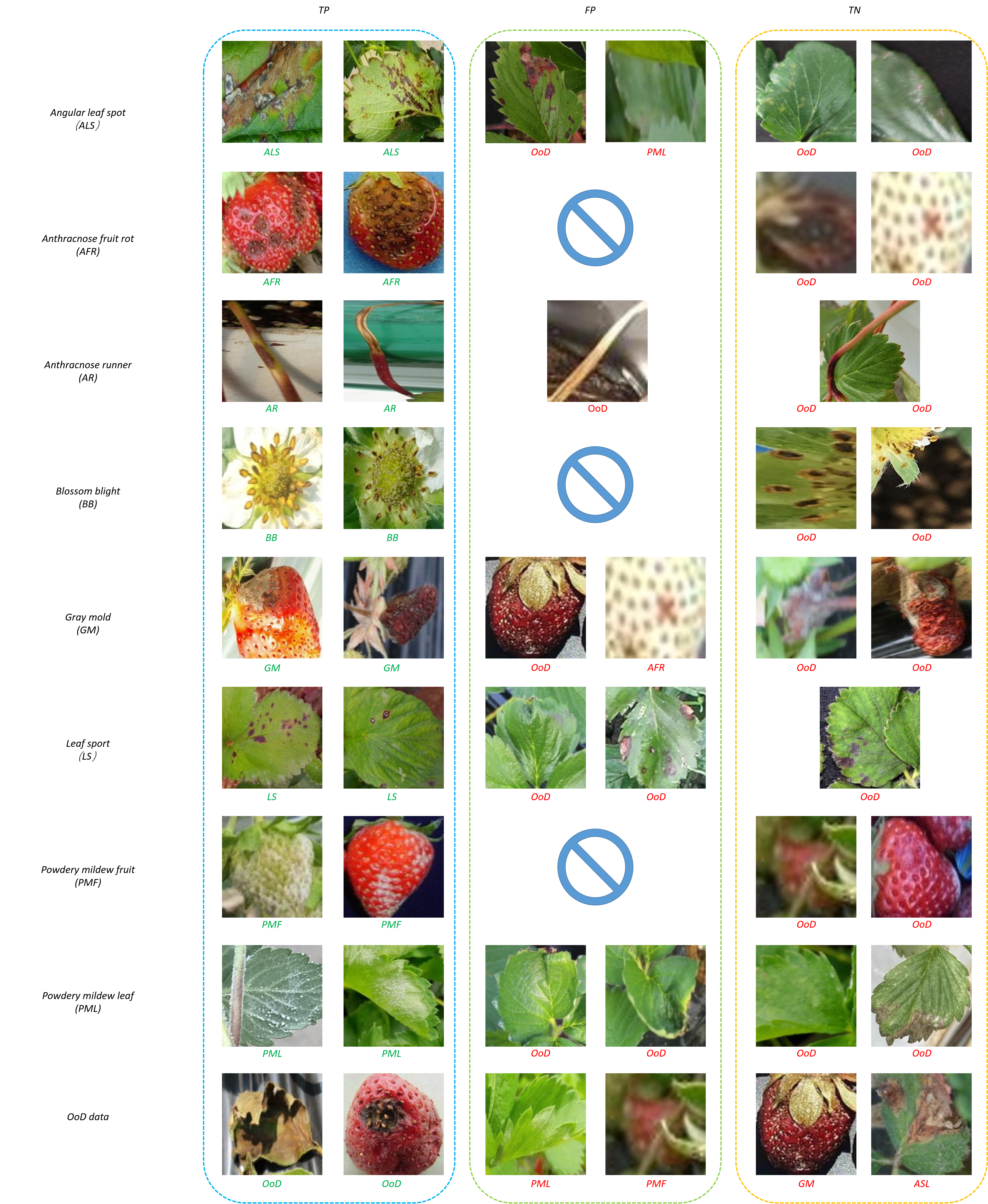

Supplement: Supplementary file 3 [file Data_Sheet_3.ZIP › fig13.png]

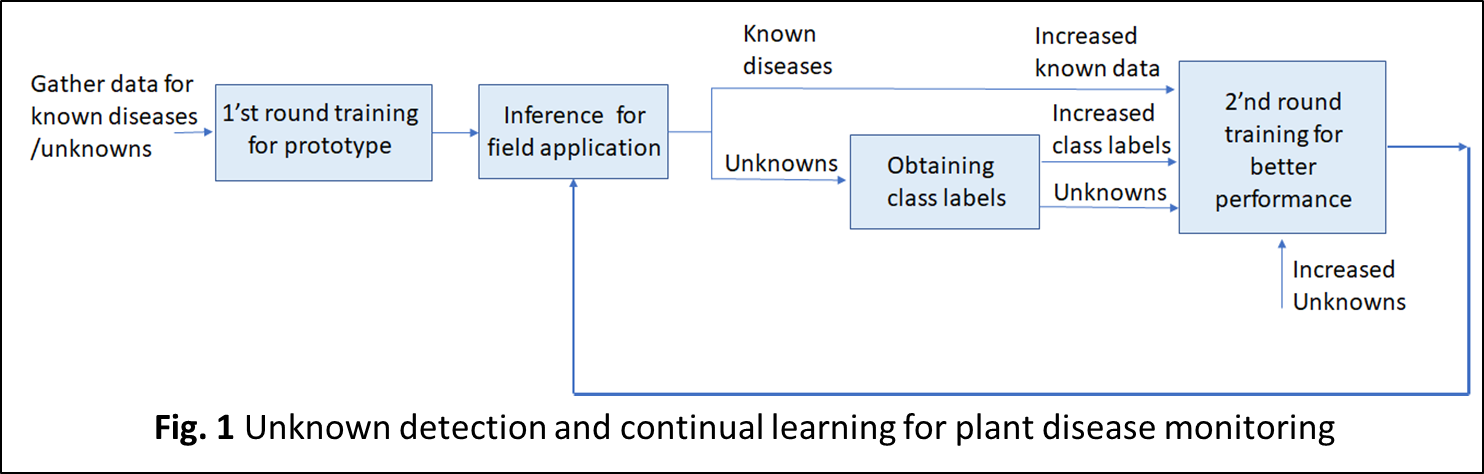

Supplement: Supplementary file 4 [file Data_Sheet_4.ZIP › fig1.png]

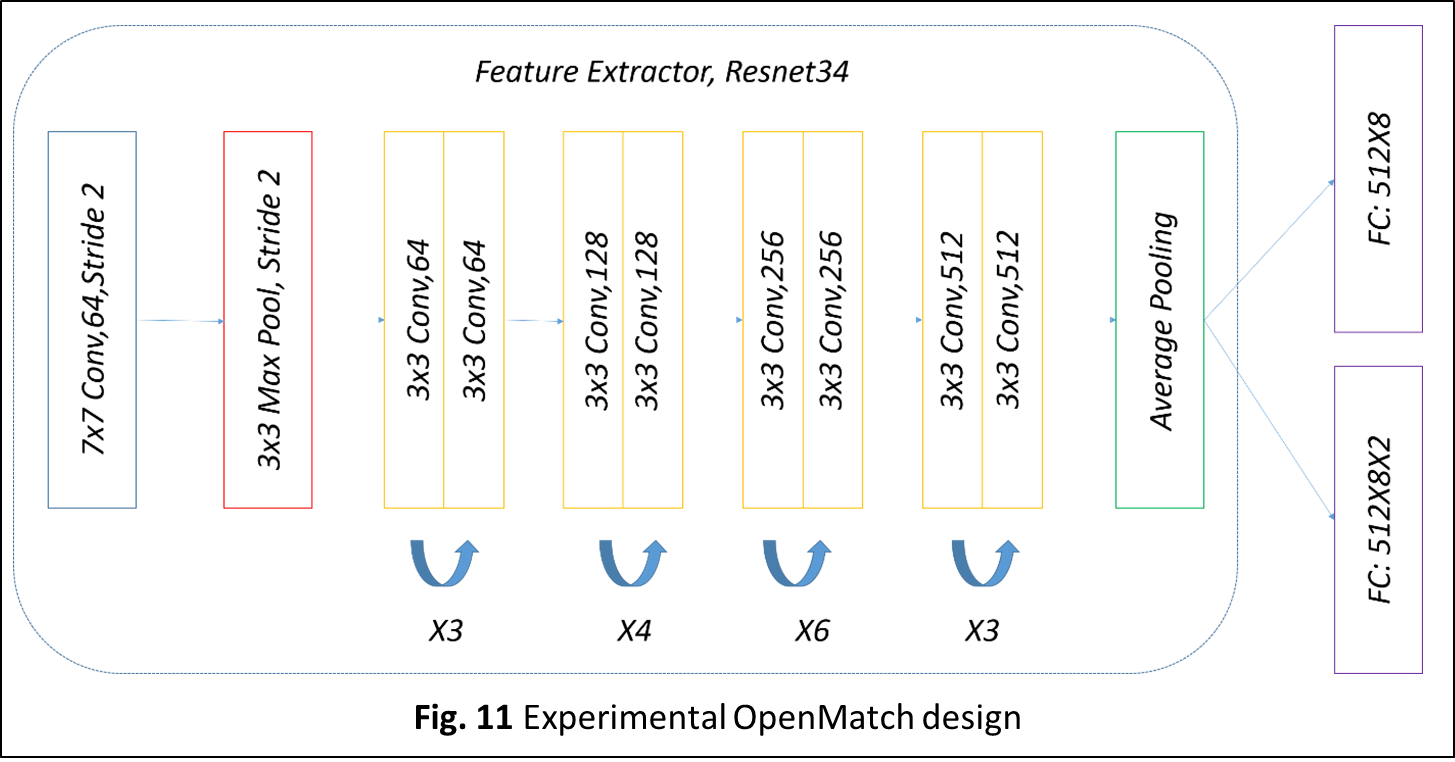

Supplement: Supplementary file 4 [file Data_Sheet_4.ZIP › fig11.png]

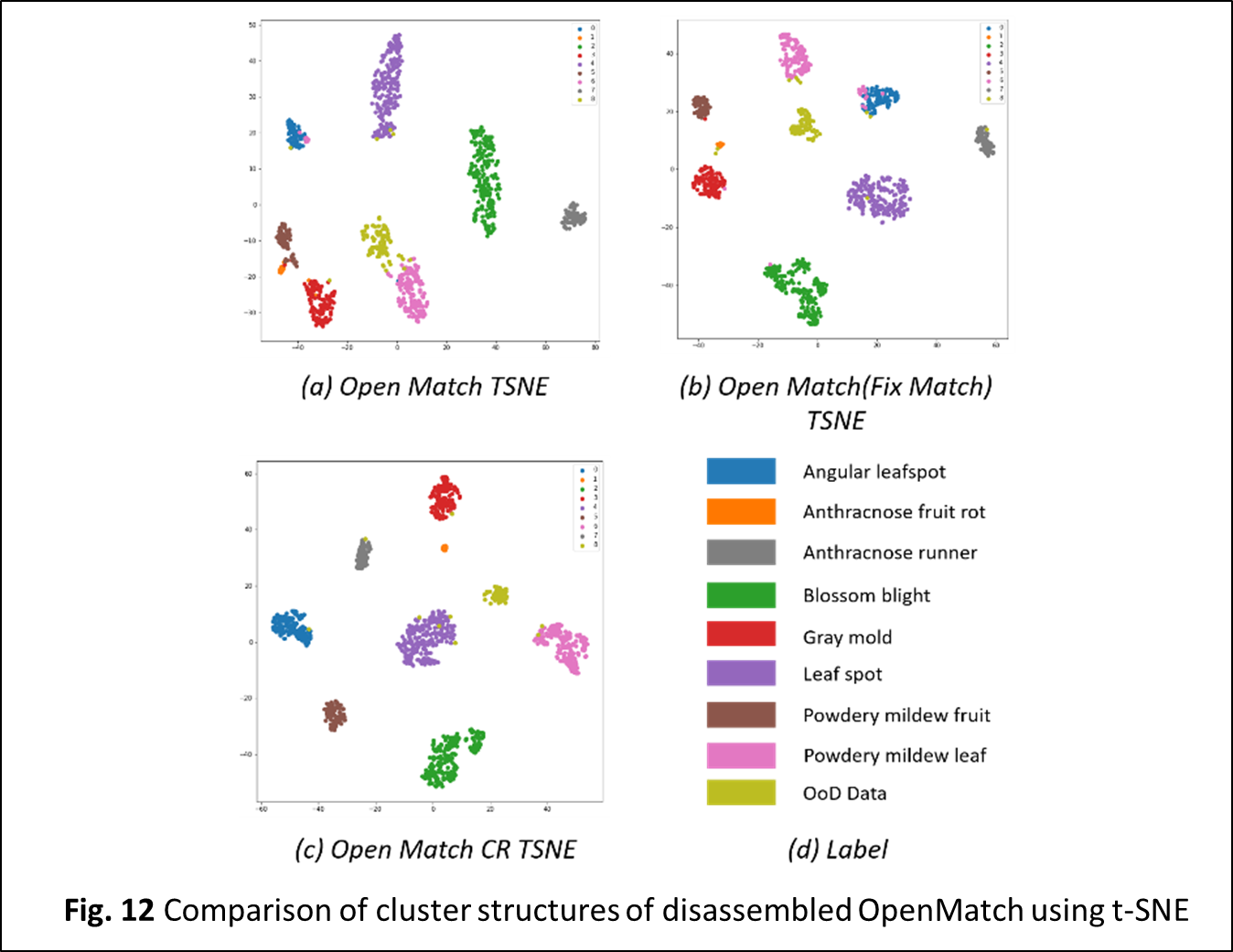

Supplement: Supplementary file 4 [file Data_Sheet_4.ZIP › fig12.png]

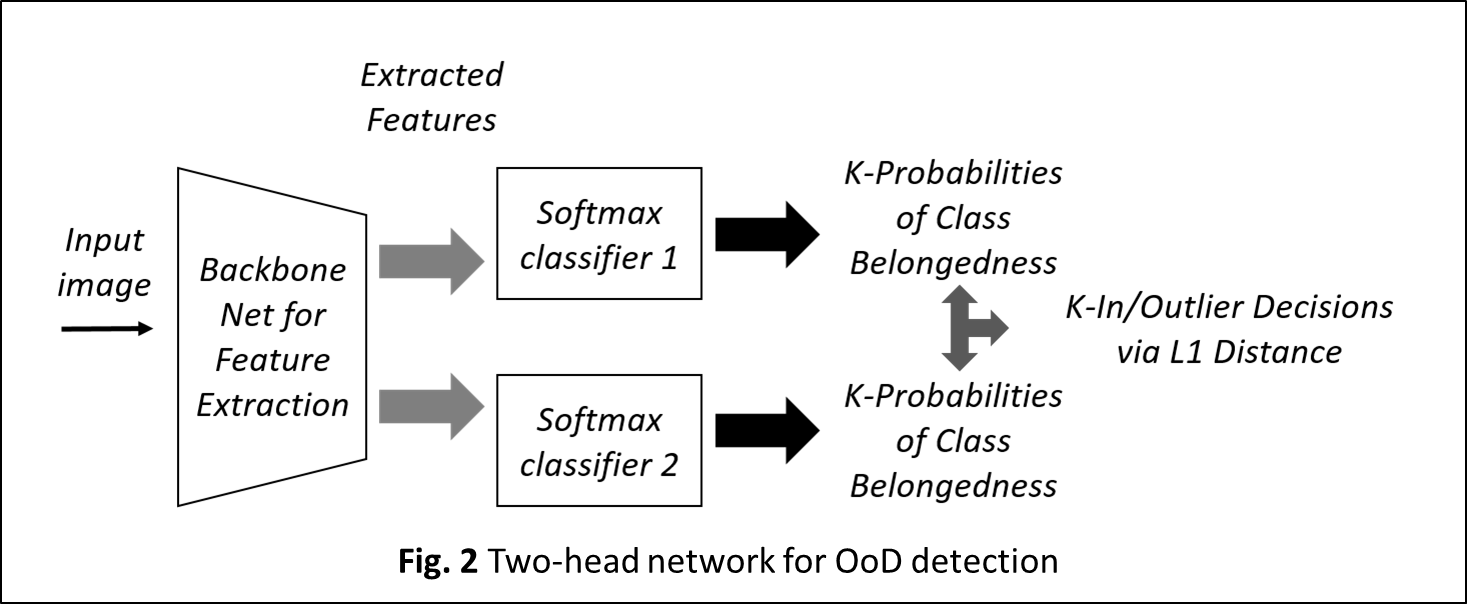

Supplement: Supplementary file 4 [file Data_Sheet_4.ZIP › fig2.png]

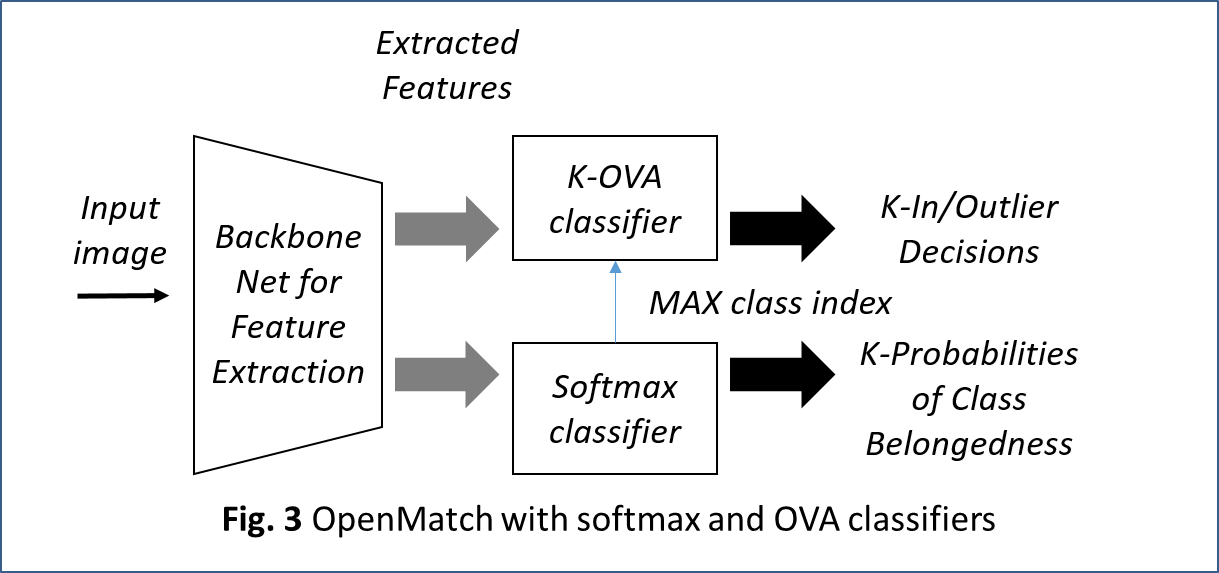

Supplement: Supplementary file 4 [file Data_Sheet_4.ZIP › fig3.png]

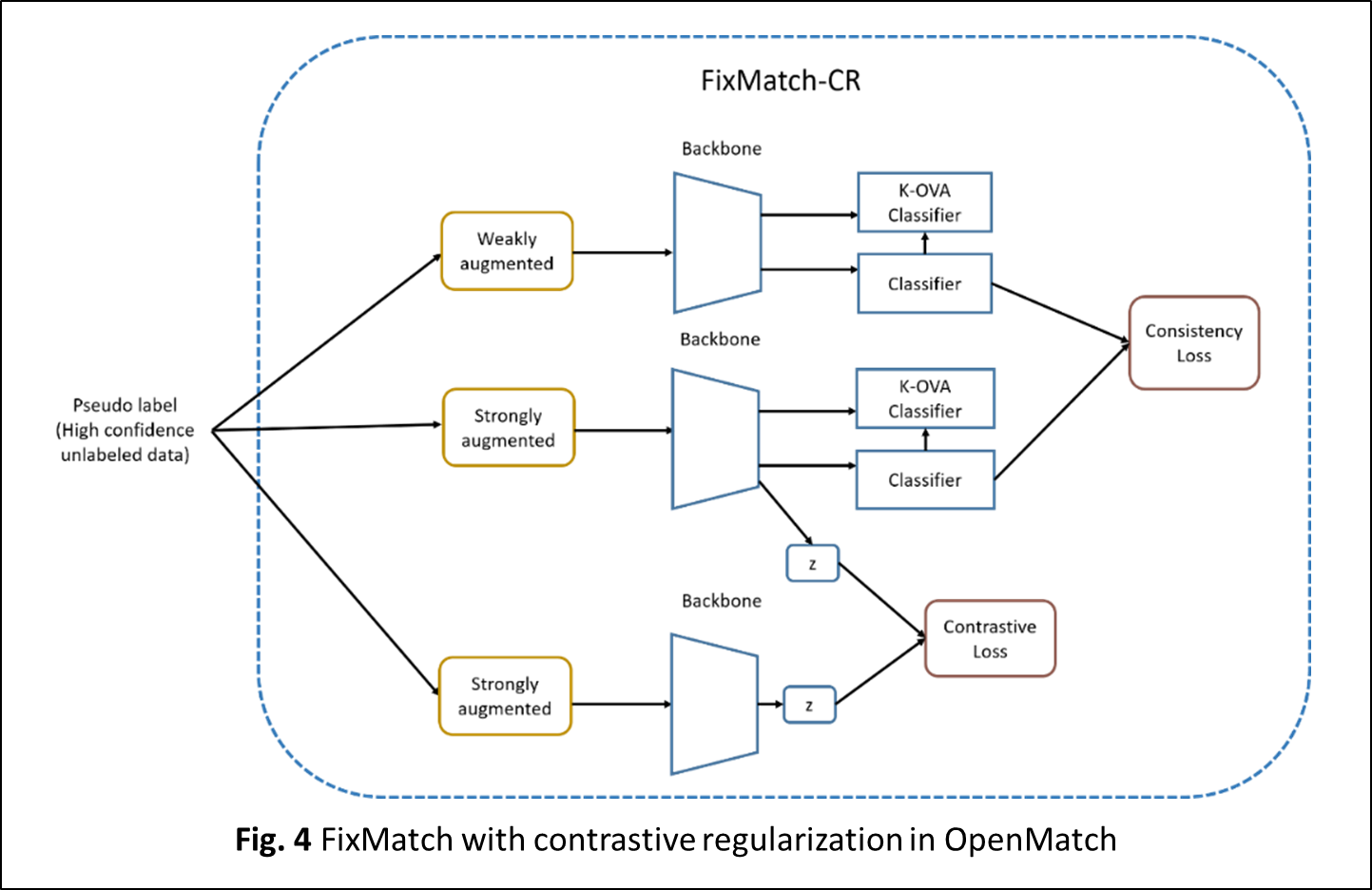

Supplement: Supplementary file 4 [file Data_Sheet_4.ZIP › fig4.png]

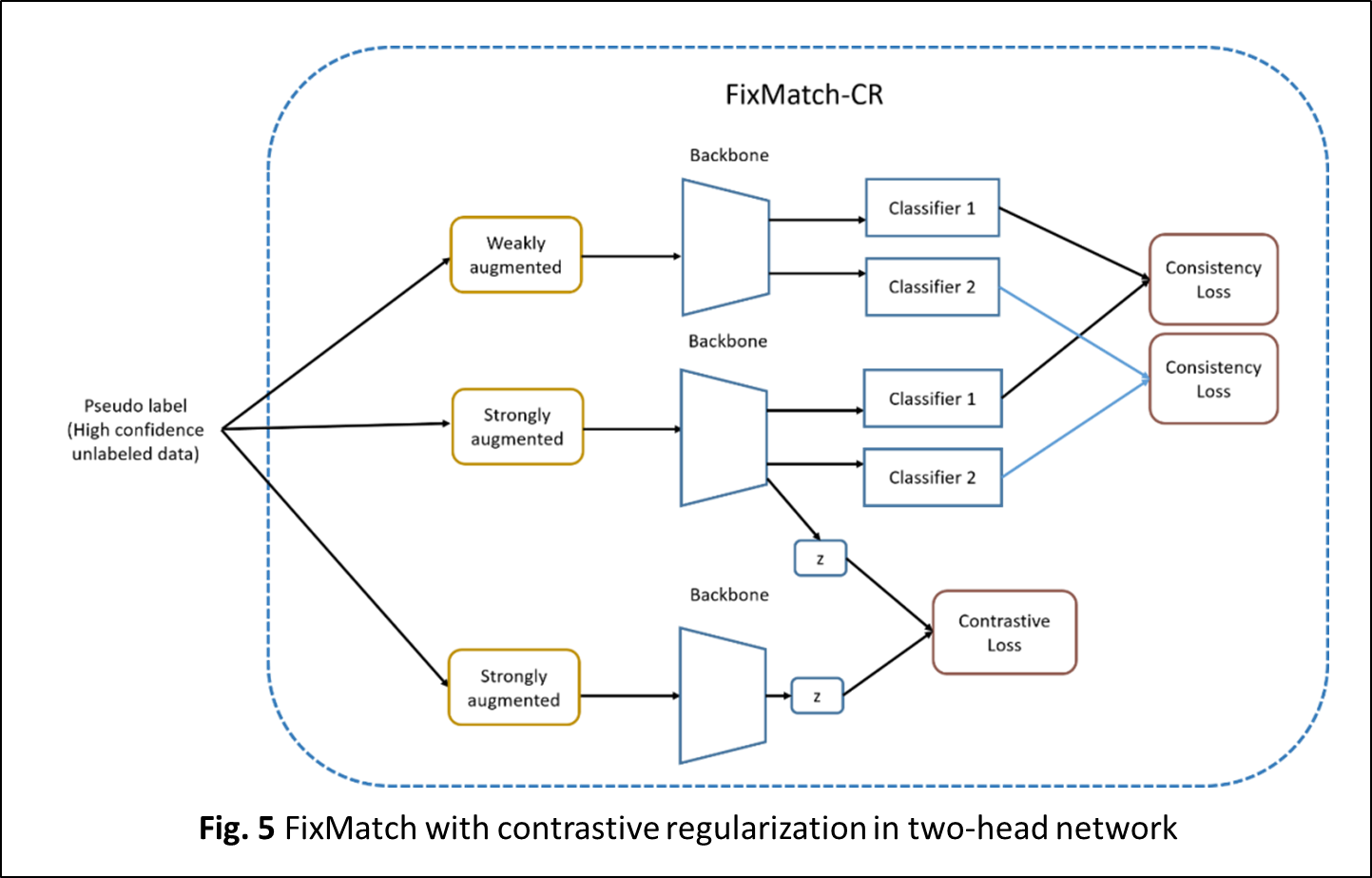

Supplement: Supplementary file 4 [file Data_Sheet_4.ZIP › fig5.png]

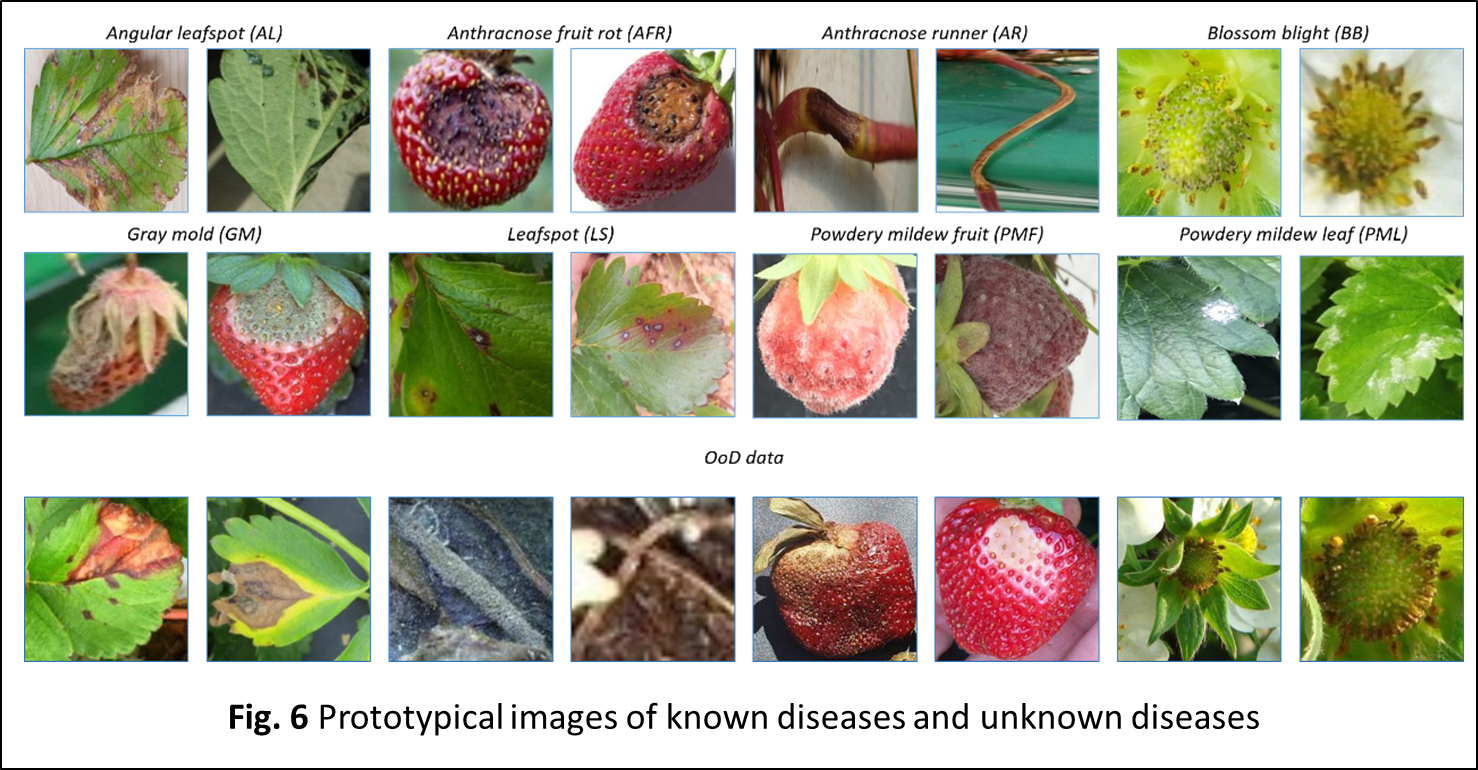

Supplement: Supplementary file 4 [file Data_Sheet_4.ZIP › fig6.png]

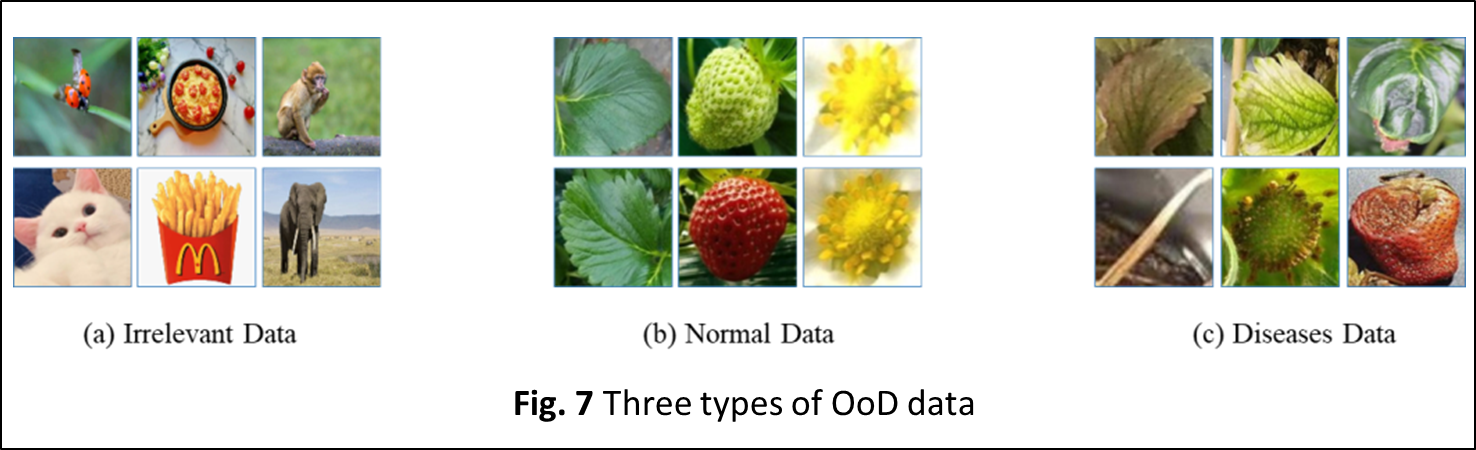

Supplement: Supplementary file 4 [file Data_Sheet_4.ZIP › fig7.png]

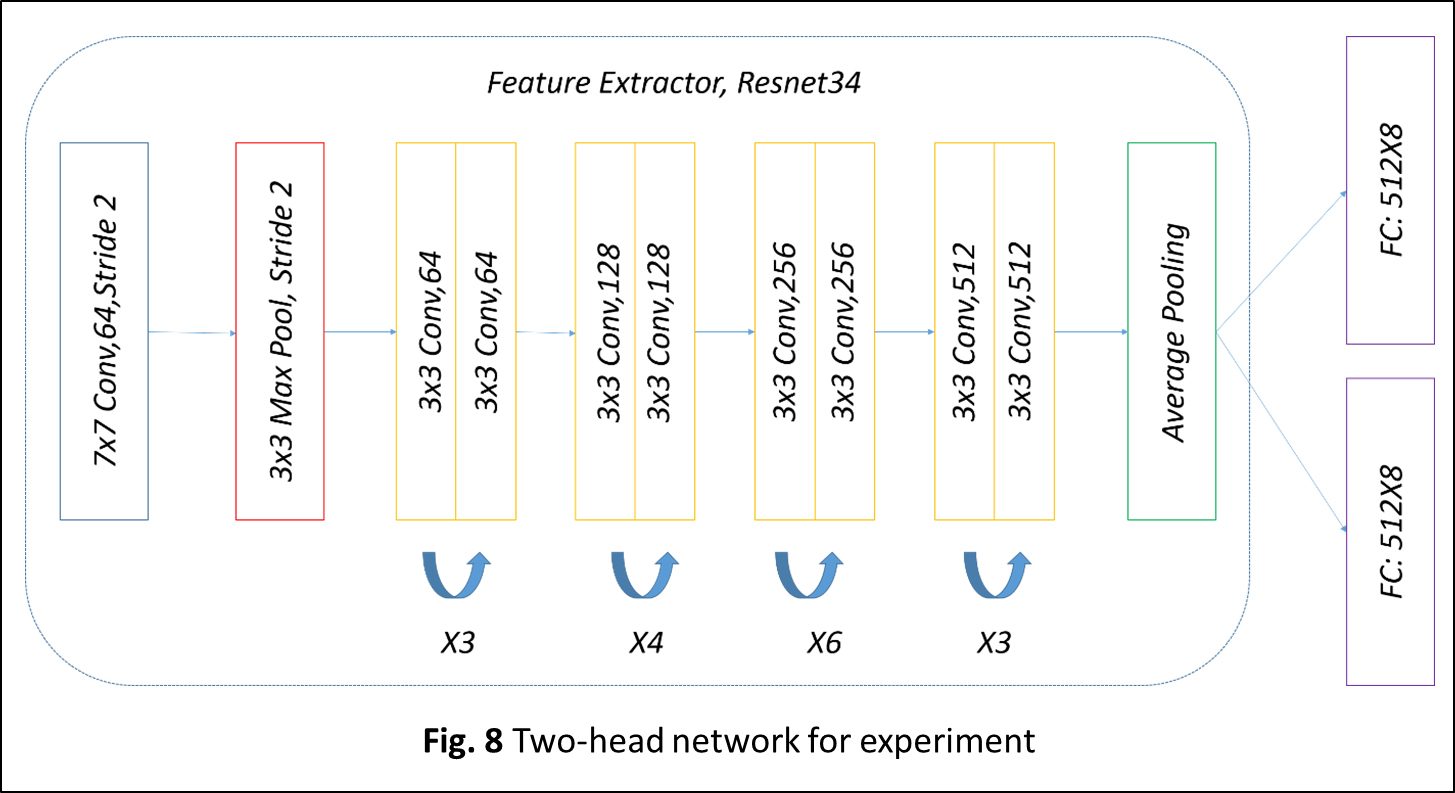

Supplement: Supplementary file 4 [file Data_Sheet_4.ZIP › fig8.png]

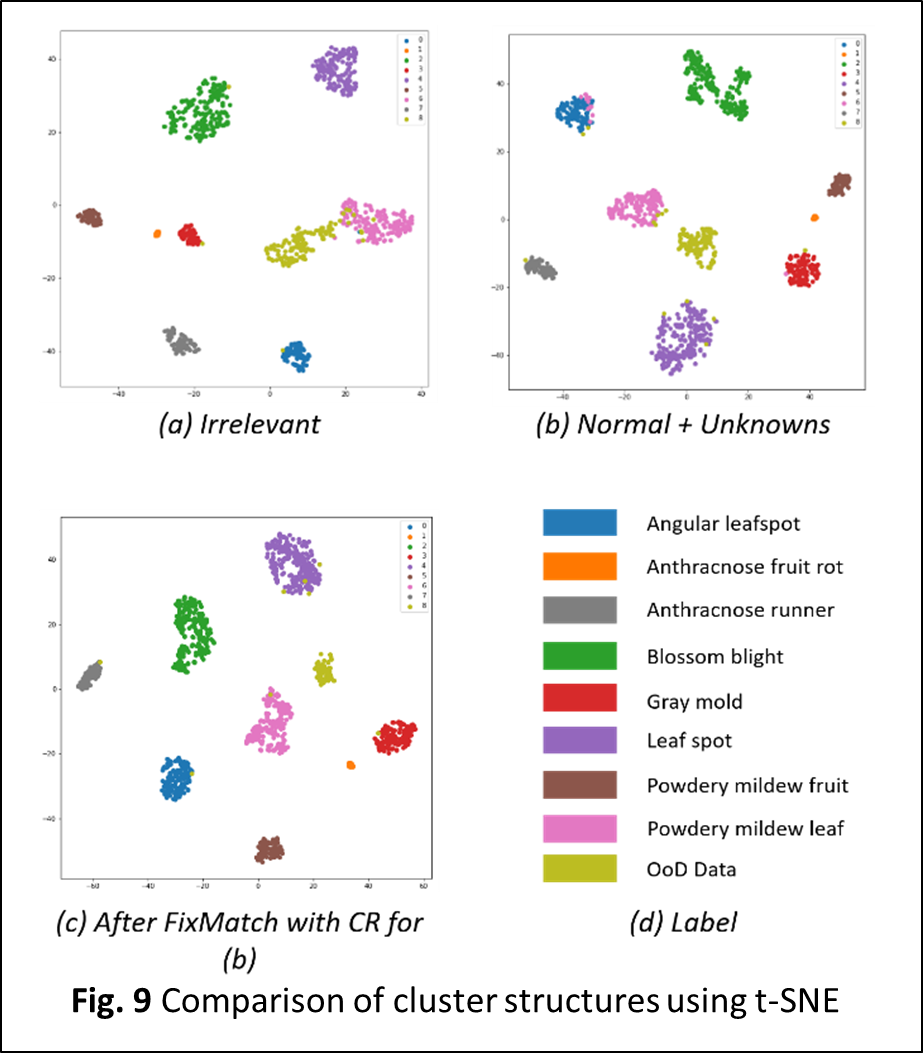

Supplement: Supplementary file 4 [file Data_Sheet_4.ZIP › fig9.png]
